# The Agassiz’s desert tortoise genome provides a resource for the conservation of a threatened species

**DOI:** 10.1371/journal.pone.0177708

**Published:** 2017-05-31

**Authors:** Marc Tollis, Dale F. DeNardo, John A. Cornelius, Greer A. Dolby, Taylor Edwards, Brian T. Henen, Alice E. Karl, Robert W. Murphy, Kenro Kusumi

**Affiliations:** 1 School of Life Sciences, Arizona State University, Tempe, Arizona, United States of America; 2 University of Arizona Genetics Core, University of Arizona, Tucson, Arizona, United States of America; 3 Natural Resources and Environmental Affairs, Marine Air Ground Task Force Training Command, Marine Corps Air Ground Combat Center, Twentynine Palms, California, United States of America; 4 Alice E. Karl and Associates, Davis, California, United States of America; 5 Centre for Biodiversity and Conservation Biology, Royal Ontario Museum, Toronto, Canada; Oregon State University Department of Botany and Plant Pathology, UNITED STATES

## Abstract

Agassiz’s desert tortoise (*Gopherus agassizii*) is a long-lived species native to the Mojave Desert and is listed as threatened under the US Endangered Species Act. To aid conservation efforts for preserving the genetic diversity of this species, we generated a whole genome reference sequence with an annotation based on deep transcriptome sequences of adult skeletal muscle, lung, brain, and blood. The draft genome assembly for *G*. *agassizii* has a scaffold N50 length of 252 kbp and a total length of 2.4 Gbp. Genome annotation reveals 20,172 protein-coding genes in the *G*. *agassizii* assembly, and that gene structure is more similar to chicken than other turtles. We provide a series of comparative analyses demonstrating (1) that turtles are among the slowest-evolving genome-enabled reptiles, (2) amino acid changes in genes controlling desert tortoise traits such as shell development, longevity and osmoregulation, and (3) fixed variants across the *Gopherus* species complex in genes related to desert adaptations, including circadian rhythm and innate immune response. This *G*. *agassizii* genome reference and annotation is the first such resource for any tortoise, and will serve as a foundation for future analysis of the genetic basis of adaptations to the desert environment, allow for investigation into genomic factors affecting tortoise health, disease and longevity, and serve as a valuable resource for additional studies in this species complex.

## Introduction

Species at risk of extinction can benefit from human interventions, and effective management of threatened populations depends on an understanding of their genetic variation, including the effects of inbreeding, disease, and adaptation to local environments [1]. One of six species of desert tortoise estimated to have arisen in North America ~35 million years ago (Ma) [2], Agassiz’s desert tortoise (*Gopherus agassizii*) has been heavily impacted by habitat loss, a respiratory tract disease [3,4], and other anthropogenic factors [5]. For instance, in one area of the species’ range density declined from about 225 individuals/km² in 1979 to about 75 individuals/km² in 1992 [6]. Such declines prompted populations of *G*. *agassizii* north and west of the Colorado River to be listed as threatened under the U.S. Endangered Species Act [5]. Despite this protection, desert tortoise numbers continued to decline ~50% between 2004 and 2013 [7], warranting further conservation efforts. Effective management of *G*. *agassizii* populations would benefit from genomic resources, which have only recently been developed for the species.

Previous population genetic studies based on microsatellites, nucleotide sequences from mitochondrial and nuclear DNA, and RNA sequencing (RNA-Seq) data have laid the groundwork for assessing the distribution of genetic diversity across the range of *G*. *agassizii* [8–10]. Agassiz’s desert tortoise is a long-lived species that is slow to mature, with a maximum lifespan of over 50 years in the wild [11], making the long-term impacts on adult populations and conservation priorities difficult to assess. Genomic analyses of *G*. *agassizii* may help to understand key aspects necessary to its survival, such as immune system responses to diseases, including upper respiratory tract disease [3]. Functional trait analysis combined with genomics can advance knowledge of physiological [12], morphological [13], and life history variation among the six species of *Gopherus* [2]. Genomic information can also inform management decisions about habitat corridors and reproduction especially in the light of climate change [14]. A whole blood transcriptome assembly has been published for *G*. *agassizii* [8]. However, this represents only a portion of the total genetic information, and a complete genome assembly would better facilitate future analyses of polymorphism and the geographic distribution of variation for the species and its congeners.

In addition to providing resources that can save threatened or endangered species from extinction, comparative genomics may reveal information about many traits relevant to human illnesses [15] and longevity [16]. *Gopherus agassizii* possesses many traits common to turtles, or chelonians, including longevity, as well as those specific to desert tortoises such as robustness to harsh environments. Genome assemblies exist for several chelonians: the Chinese softshell turtle, *Pelodiscus sinensis* [17], the green sea turtle, *Chelonia mydas* [17], and the western painted turtle, *Chrysemys picta bellii* [18]. Embryonic transcriptomes have been published for the red-eared slider turtle, *Trachemys scripta* [19], a blood transcriptome for the giant Galapagos tortoise, *Chelonoidis nigra* [20], and liver transcriptomes for the common musk turtle (*Sternotherus odoratus*), common snapping turtle (*Chelydra serpentina*), and African sideneck turtle (*Pelusios castaneus*) [21]. Recent studies have confirmed a sister-taxon relationship between chelonians (including turtles and tortoises, order Testudines) and archosaurs (including crocodilians and birds), which split ~250 Ma [17,18], and have estimated that the basal divergence of most modern chelonian lineages occurred ~100 Ma. *Gopherus agassizii* and *C*. *picta bellii* last shared a common ancestor ~70 Ma [22] (Table 1), and comparisons using this more recent divergence may provide a better opportunity to understand the evolution of many chelonian-specific traits, such as longevity and adaptations to aquatic or terrestrial habitats, including hot deserts. To assist both conservation and comparative genomics, we report a whole genome assembly and deep transcriptomic sequence-based annotation for Agassiz’s desert tortoise, and provide insights from comparative and population genomic analyses.

**Table 1:** Chelonian species with whole genome sequences.

Scientific Name	Family	Common Name	Citation
*Gopherus agassizii*	Testudinae	Agassiz’s desert tortoise	Current study
*Chrysemys picta bellii*	Emydidae	Western painted turtle	[18]
*Chelonia mydas*	Cheloniidae	Green sea turtle	[17]
*Pelodiscus sinensis*	Trionynichidae	Chinese softshell turtle	[17]

## Materials and methods

### Sample collection and sequencing

The Institutional Animal Care and Use Committee at Arizona State University reviewed and approved this study, and methods of transport, housing, and euthanasia were carried out in accordance with ethical guidelines in Protocol #13-1319R (to DFD). Specifically, an adult male *G*. *agassizii* collected in urban Las Vegas, Nevada under U.S. Fish and Wildlife Service recovery subpermit #FWSDTRO-1 was transported to Arizona under Nevada Department of Wildlife export permit #S37016, housed for two days and euthanized with an intracoelemic injection of sodium pentobarbital and phenytoin solution. Tissues were harvested under sterile conditions and either flash-frozen in liquid nitrogen or placed in RNAlater (Thermo Fisher Scientific) for DNA and total RNA extraction, respectively. DNA was extracted from liver and skeletal muscle using the DNeasy kit (QIAGEN) and total RNA was isolated from lung, brain and skeletal muscle with the total RNA protocol included in the mirVana miRNA Isolation Kit (Thermo Fisher Scientific). RNA quantity and quality was measured by spectrophotometry (Nanodrop) and Agilent 2100 Bioanalyzer. All sequencing was carried out on the Illumina HiSeq 2000 or 2500 platforms. Paired-end DNA libraries were constructed with targeted fragment sizes of 200 base-pairs (bp), 300 bp, and 1,000 bp using the Kapa Biosystems Library Prep Kit by the Collaborative Sequencing Center at the Translational Genomics Research Institute (Phoenix, AZ; S1A Table). Mate-pair libraries were constructed and sequenced for DNA fragment sizes of 2 kbp, 4 kbp, and 10 kbp (S1 Table). Additionally, RNA-Seq libraries for brain, muscle and lung total RNAs were constructed and sequenced at the University of Arizona Genetics Core (Tucson, AZ; S1B Table).

To confirm the taxonomic identity of the specimen, and to assure that it was not a hybrid of *G*. *agassizii* and *G*. *morafkai* [23], we followed described protocols [23] using 25 short tandem repeats and mtDNA sequence data for population assignment against a database of 1,258 specimens of *Gopherus*. We confirmed the specimen to be *G*. *agassizii* from the northern Mojave genetic unit, which is geographically bounded as east of the Ivanpah, California area and northwest of the Colorado River in Nevada, Arizona and Utah [12].

### Genome assembly

DNA sequence reads were trimmed to eliminate nucleotide biases and remove adaptors with Trimmomatic v0.27 [24]. We retained all reads ≥ 37 bp with a quality score ≥ 28. Illumina sequencing errors were corrected using SOAPec v2.01, and overlapping reads from the 200 bp libraries were joined to form single-end reads using FLASH v1.2.8 [25]. We compared the outputs of different de Bruijn graph assemblers, including ABySS 1.5.2 [26], SOAPdenovo2 [27], and Platanus v1.2.1 [28] for both contig and scaffold assembly, in addition to SSPACE v3.0 [29] for scaffold assembly; we then closed gaps using the GapCloser v1.12 module from SOAP (S1 Appendix). The assemblies with the most scaffold contiguity (i.e., N50) and reasonable total length given the expected genome size were selected for further analysis. We evaluated completeness of the assemblies by their estimated gene content, using the Conserved Eukaryotic Genes Mapping Approach (CEGMA v2.5) [30], which calculated the proportion of 248 core eukaryotic genes present in the genome assembly, and Benchmarking Universal Single Copy Orthologs (BUSCO v1.22) [31], which calculated the proportion of a vertebrate-specific set of 3,023 conserved genes that were either complete, fragmented, or missing. We further improved the raw assembly by RNA scaffolding. In brief, assembled transcripts (described below) were searched for open reading frames (ORFs) and filtered to include only sequences that produced a significant BLAST hit to a protein sequence in the UniProtKB/Swiss-Prot database [32] using TransDecoder v2.0 [33] (https://transdecoder.github.io/). This filtered gene set was mapped to the genome assembly using BLAT [34] and identified scaffolds were merged using L_RNA_scaffolder [35].

### Genome annotation

The RNA-Seq fastq files for skeletal muscle, brain and lung were filtered for adapters, quality score ≥ 30 and length ≥ 37 bp using Trimmomatic v0.27, and paired-end and single-end reads were assembled together into transcripts using Trinity v2.0.6 [36]. Previously published whole blood-based transcriptome data were also used (NCBI BioProject PRJNA281763) [8]. We used MAKER2 v2.31.8 [37] in multiple iterations for gene-finding, which incorporated: (1) direct evidence from the transcriptomes, (2) homology to proteins in the UniProtKB/Swiss-Prot database and the predicted proteome of *C*. *picta bellii* (NCBI BioProject PRJNA210179), and (3) *ab initio* predictions from SNAP (11/29/2013 release) [38] and Augustus v3.0.2 [39]. The first iteration of MAKER aligned the transcript and protein sequences to the assembly, producing draft gene models. *Ab initio* gene predictors benefit from the training of their Hidden Markov Models (HMM) to the species-specific genome, and we trained SNAP using a two-pass approach in MAKER. First, we ran MAKER a second time with SNAP using *G*. *agassizii*-specific HMMs generated from the initial MAKER iteration. Using the gene models from this output, we then generated an improved HMM for SNAP in a third MAKER iteration. In parallel, Augustus was trained by sampling the SNAP-improved MAKER gene models followed by optimization. A fourth and final run of MAKER was then performed, which incorporated the optimized HMM for Augustus, the final SNAP HMM, and the aligned protein and transcriptome data, resulting in the final set of gene model predictions.

Gene predictions were functionally annotated by collecting the top hits from a BLASTP search of the UniProtKB/Swiss-Prot database. Protein predictions from the final gene models were assigned one-to-one orthology with proteins from the western painted turtle genome through reciprocal BLAST. First, BLASTP was used to search for the best hit for every predicted Agassiz’s desert tortoise protein in the western painted turtle proteome (evalue = 1E-6, max_target_seqs = 1), followed by BLASTP of each western painted turtle protein in the Agassiz’s desert tortoise proteome. We also used reciprocal best hits to identify orthologs of Agassiz’s desert tortoise in Chinese softshell turtle, chicken (*Gallus gallus*), green anole (*Anolis carolinensis*), and human proteomes. Clusters of homologous genes across Agassiz’s desert tortoise, western painted turtle, Chinese softshell turtle, chicken, American alligator (*Alligator mississippiensis*), green anole and human were identified using OrthoFinder v0.4 [40].

### Repeat analysis

The Agassiz’s desert tortoise genome assembly was masked for repetitive elements, first with RepeatMasker v4.0.5 [41] using the complete library of known repeats available in RepBase [42]. The remaining unmasked regions of the desert tortoise genome were then searched for repeat families using RepeatModeler v1.0.8 [43]. This output of *de novo* repeats from desert tortoise was then combined with the first RepBase library to create a combined library, which was used in a final repeat-masking step to classify all annotated repeats in the genome. We also ran RepeatMasker on the western painted turtle genome using RepBase, which includes repeat libraries specific to *Chrysemys picta bellii*, in parallel for comparison to Agassiz’s desert tortoise. To estimate the amount of evolutionary divergence within repeat families of Agassiz’s desert tortoise, we generated repeat-family-specific alignments and calculated the average Kimura 2-parameter (K2P) distance from consensus within each family, correcting for high mutation rates at CpG sites with the calcDivergenceFromAlign.pl tool included with RepeatMasker. For comparison, the divergence profile for western painted turtle was obtained from the RepeatMasker server (http://www.repeatmasker.org/species/chrPic.html, accessed August 10, 2015).

### Comparative genomic analyses

To understand patterns of molecular evolution across chelonians and other reptiles, the genome assemblies of Agassiz’s desert tortoise, western painted turtle, Chinese softshell turtle, green sea turtle, American alligator (allMis1), budgerigar (*Melopsittacus undulatus*, melUnd1), and green anole lizard (AnoCar2.0) were aligned to the reference chicken genome (galGal4) using LASTZ v1.0.2 [44]. We used chaining to form gapless blocks and netting to select the highest-ranked chains [45]. Pairwise alignments for zebra finch (*Taenopygia guttata*, taeGut3) and medium ground finch (*Geospiza fortis*, geoFor1) to chicken were downloaded from the UCSC Genome Browser. A guide tree representing the phylogenetic relationships of the included taxa [46,47] was used to construct a 10-way whole genome alignment (WGA) from the pairwise alignments with MULTIZ v11.2 [48]. The WGA was filtered to include alignment blocks containing at least nine out of the 10 species.

Divergence at fourfold degenerate (4D) sites, which approximates the neutral mutation rate [49], was used to compare variation in DNA substitution rates of chelonians and the other sauropsids. We extracted 4D sites from the WGA based on the Ensembl v82 chicken gene annotations using msa_view in the PHAST v1.3 package [50]. To reconstruct the phylogeny of the 10 sauropsids included in the WGA using the 4D data, we used RAxML v8.2.3 [51]. We generated 20 maximum likelihood (ML) trees under the GTRCAT substitution model and conducted 500 bootstrap replicates to assess statistical support for the ML tree with the highest likelihood. We used the topology of the best ML tree from RAxML and obtained branch lengths in terms of substitutions per site by fitting a time-reversible model (REV) to the 4D site data using phyloFit in PHAST. We then estimated lineage-specific substitution rates in sauropsids using the penalized likelihood method implemented in r8s v1.8.1 [52] while placing constraints on nodal ages obtained from TimeTree [53]. The age of the ancestral sauropsids node was fixed at 280 Ma and the ancestral archosaur node at 245 Ma; these were the median age estimates. Estimates of divergence time for most chelonian families differed widely in the literature. Thus, we placed minimum and maximum age constraints on the nodes representing Cryptodira (encompassing all four represented chelonian families, 97.4 Ma to 250 Ma) and Drurocryptodira (excluding *P*. *sinensis*, 36 Ma to 183 Ma). The divergence time of Testudinae (*G*. *agassizii*) and Emydidae (*C*. *picta bellii*) was estimated (1) without constraint and (2) with constraints, which fixed the node age as the median estimate from TimeTree (74.2 Ma).

To identify protein-coding genes potentially responsible for adaptations in Agassiz’s desert tortoise, we calculated the ratio of non-synonymous to synonymous substitutions (Ka/Ks) in pairwise comparisons separately to western painted turtle (chrPic2), Chinese softshell turtle (pelSin1), green sea turtle (cheMyd1) and chicken. Syntenic genome alignments were generated as described above, and the Stitch Gene Blocks tool in Galaxy [54] was used to extract orthologs using the chicken reference annotation (Ensembl v82). We removed gene alignments with less than 50% coverage, ambiguous codons, and internal stop codons using AlignmentProcessor (https://github.com/WilsonSayresLab/AlignmentProcessor). Estimated Ka/Ks for the filtered gene alignments were obtained using KaKs_calculator2.0 [55], and we collected the RefSeq (western painted turtle, green sea turtle, Chinese softshell turtle) or Ensembl (chicken) IDs of accelerated genes (Ka/Ks > 1). These genes were enriched for non-synonymous and potentially functional substitutions resulting from amino acid changes [56]. A Bonferroni adjustment for multiple hypothesis testing [57] was used to identify genes with highly significant Ka/Ks > 1, which indicates positive selection. Many genes in chelonian genome assemblies lacked clear orthology to an organism in RefSeq. Thus, human orthologs were obtained by the ID Mapping of associated gene names in UniProt. Next, Ensembl BioMart [58] was used to for retrieve Gene Ontology (GO) terms. REVIGO [59] semantic clustering was used to visualize GO terms for biological processes, cellular components and molecular function. To compare GO terms, analyses used medium similarity (0.7) for semantic overlap, clustering of terms by ‘uniqueness’ and the default whole UniProt database.

In 2011, divergence across genetics, ecology and behavior was used to recognize two different species of desert tortoise in the southwestern United States: Agassiz’s desert tortoise (*G*. *agassizii*), found in the Mojave Desert of California, Nevada, Utah and northwestern Arizona, and Morafka’s desert tortoise (*G*. *morafkai*), found in the Sonoran Desert of Arizona and Sonora, Mexico [60]. More recently, genetic data were used to characterize and describe the third desert species, Goode’s Thornscrub tortoise (*G*. *evgoodei*), which resides in the states of Sonora and Sinaloa, Mexico [61]. These three species are thought to have diverged nearly simultaneously ~5.5 Ma [8] and occupy diverse habitats, including the high desert of the Great Basin shrub steppe in Utah, to the Joshua tree forests of California, through the coastal desertscrub along the east coast of the Sea of Cortez and the lush tropical deciduous forests of Sinaloa, Mexico [62]. The three species of desert tortoise have likely adapted to different climatic regimes and landscapes. To find genomic regions responsible for species-specific desert tortoise adaptations, we analyzed fixed unique variants within three species of the desert tortoise species complex. We mapped previously published blood RNA (NCBI BioProject PRJNA281763) data from three individuals per species to our new *G*. *agassizii* reference genome using STAR v.2.5.0a [63], with default parameters for the two-pass method set to “basic” to find splice junctions. We added read groups and removed PCR duplicates using MarkDuplicates from Picard v1.140 (http://broadinstitute.github.io/picard/). We then used the Split’N’Trim tool in the Genome Analysis Tool Kit pipeline (GATK v3.4–46) [64] to split reads into exon segments and clip sequences that overlap introns. The split, de-duplicated alignment files from each individual were then merged using Picard. We used the CallableLoci GATK tool to filter the genome for areas that met default coverage criteria for each alignment file, and concatenated, sorted and merged the output BED files using bedtools version 2.24.0 [65], with–d set to 20 to join nearby yet non-contiguous callable sites. Using the merged callable loci as a target file, we called variants on all nine alignments with FreeBayes v1.0.1 [66].

We identified fixed differences between the three species using the Filter command in SnpSift v4.0 [67], filtering by quality (≥ 30) and read depth (≥ 20). We filtered for only SNP variants within the unique variants file per taxon, excluding STRs and insertion-deletions. We cross-referenced these unique SNP variant files to orthology tables for human (*Homo sapiens)*, chicken (*Gallus gallus*), and green anole (*Anolis carolinensis*) (see Genome Annotation Methods) for each *Gopherus* species (9 comparisons in total). Each set of cross-referenced orthologous Ensembl IDs were: 1) converted to Gene Ontology (GO) terms using g:Convert (http://biit.cs.ut.ee/gprofiler/gconvert.cgi, accessed July 31, 2016) [68], to plot and analyze semantically with REViGO, and 2) entered into g:GOSt [67] to test for statistical enrichment of GO terms per *Gopherus* species. For enrichment analysis in g:GOSt we retained only significant results, used hierarchical sorting without filtering, and used the default significance threshold (g:SCS) correction for multiple hypothesis testing. For visualization in REViGO, we allowed medium similarity (0.7) for semantic overlap, clustered terms by ‘uniqueness,’ and used the default whole UniProt database for comparison of GO terms. Gene clusterings and GO-term enrichments were similar across reference taxon (human, chicken, or the green anole), so we only show results using orthology assignments from the green anole.

### Accession numbers

Genomic and transcriptomic sequences are available from the NCBI BioProject (accession numbers PRJNA352725, PRJNA352726, and PRJNA281763). The genome assembly is available from NCBI BioProject PRJNA352726. Genome and transcriptome assembly, predicted protein, transcript, genome annotation, and annotated repeat files are available from the Harvard Dataverse (doi:10.7910/DVN/EH2S9K).

## Results and discussion

### Genome sequencing and assembly

**Fig 1:**
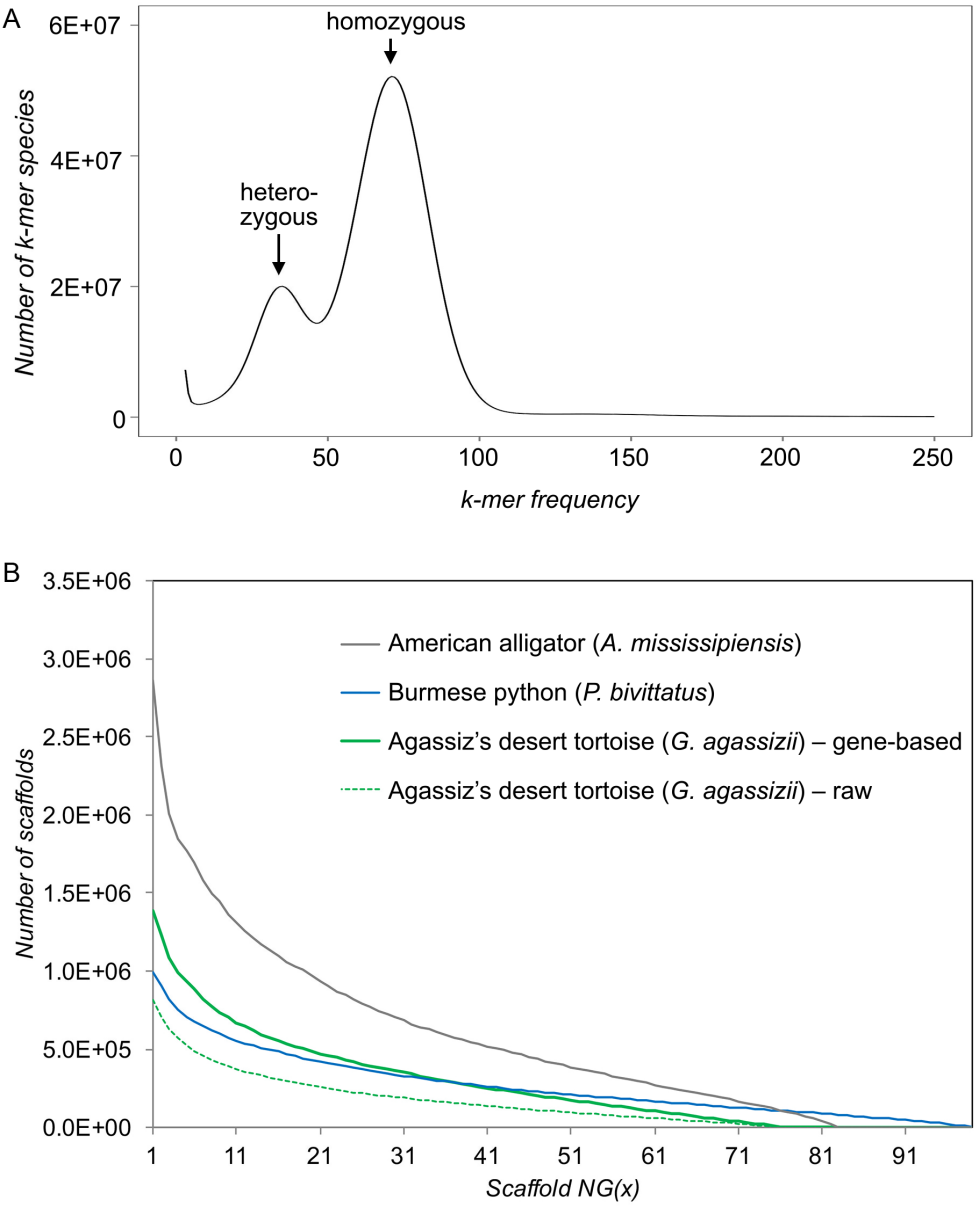
Assembly statistics for the genome of *Gopherus agassizii* and comparison with *Python bivittatus* and *Alligator mississippiensis*. (A) Two k-mer frequency peaks exist for *G*. *agassizii*: a peak at 35X coverage, representing heterozygous regions and a peak at 71X coverage, representing homozygous regions. (B) Gene-based scaffolding greatly increased scaffold N50 from 169.2 kbp to 251.6 kbp, which places it within the range of recently published genomes of reptiles including the Burmese python [71] and the American alligator [72].

For whole genome sequencing, we used multiple insert size genomic libraries (Table 2). We generated a total of 2,877,286,716 small-insert library reads with targeted insert sizes of 200 bp, 300 bp, and 1,000 bp (average insert sizes of 189 bp, 265 bp, and 788 bp, respectively). Average read length was 104 bp, and after trimming we retained 85% of those reads for an average read length of 96 bp. Using a k-mer of 27, the k-mer frequency spectrum of the trimmed short insert library data (Fig 1A) revealed peaks at 35X k-mer depth for heterozygous regions and 71X k-mer depth for homozygous regions. Using the formula *genome size = total k-mers ÷ peak k-mer depth*, we estimated the genome size for *G*. *agasizzi* to be 2,350,283,237 bp or ~2.4 gigabase-pairs (Gbp), which is smaller than a previous estimate of 2.9 Gbp based on Feulgan densitometry (FD) of red blood cells [69]. This 17% difference may reflect either error in experimental quantification or intraspecific variation in genome size. Another potential explanation for this discrepancy could be that the FD quantification of genome size was published in 1965, long before the taxonomic reclassification of the desert tortoise species complex [60,61], and thus may have been performed using a species other than *G*. *agassizii*. Using our k-mer based estimate of genome size, we further estimate that with the short insert libraries we obtained genome coverage of 128X before trimming and 97X after trimming. We also generated mate pair libraries with targeted insert sizes of 2 kbp, 4 kbp, and 10 kbp (average insert sizes of 911 bp, 2,220 bp, and 4,446 bp, respectively), with an average read length of 100 bp after trimming for additional 19X coverage. Genomic sequence summaries are listed in Table 2, and raw data is made available through NCBI BioProject PRJNA352726.

**Table 2:** Sequences used for the assembly of the Agassiz’s desert tortoise (*Gopherus agassizii*) genome.

Targeted Library Size	Number of Reads	Estimated Coverage^1^
200 bp paired-end	1,222,612,586	54X
300 bp paired-end	1,186,573,102	53X
1 kbp paired-end	468,101,028	21X
2 kbp mate pair	147,436,368	6X
4 kbp mate pair	182,973,840	8X
10 kbp mate-pair	112,067,096	5X
Total	3,319,764,020	147X

^1^Based on an estimated total genome size of 2.4 Gbp, see Results and discussion.

**Fig 2:**
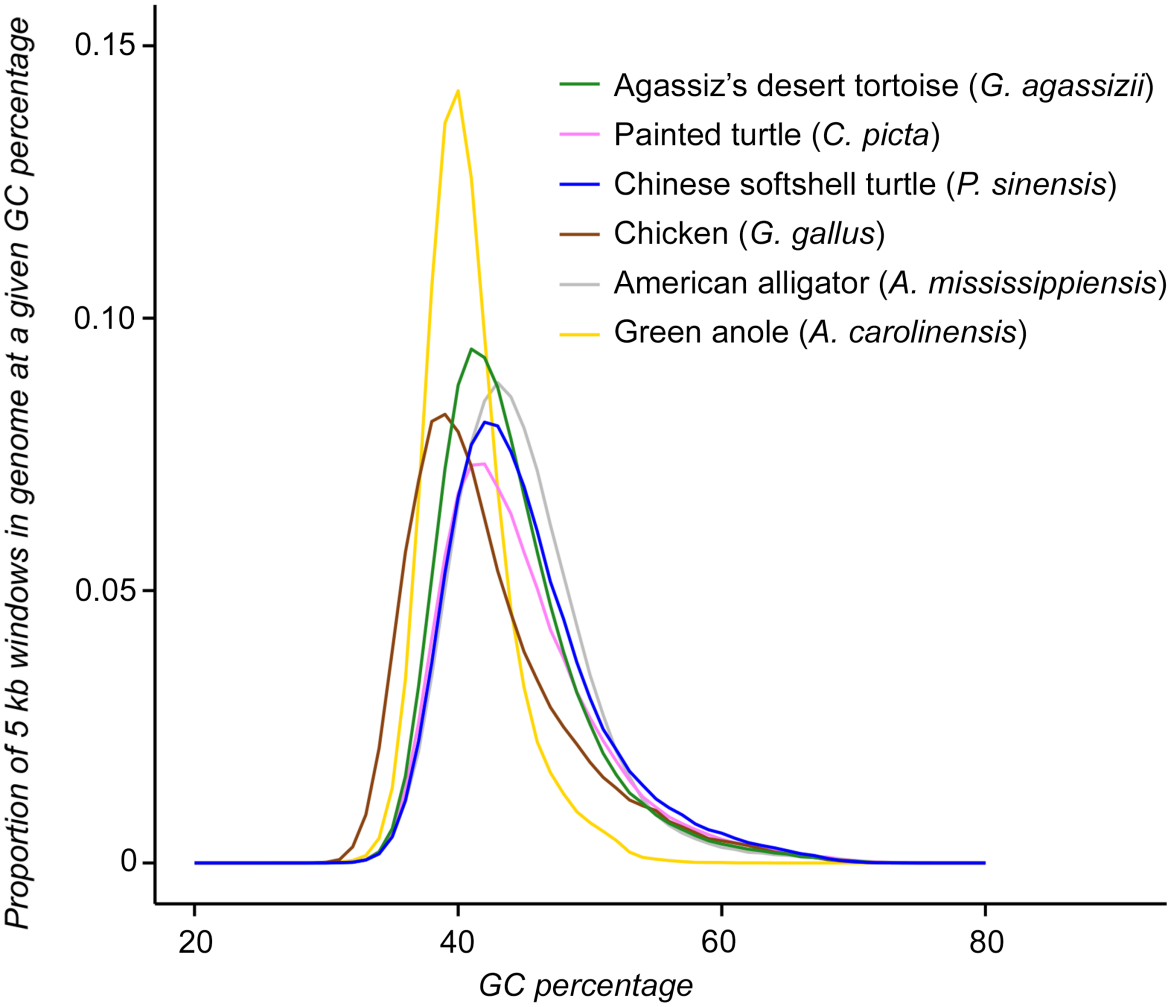
Frequency distribution (proportion) of GC content calculated in 5 kbp non-overlapping windows for *Gopherus agassizii* and other reptilian genomes. GC content of *G*. *agassizii* closely matches that of the turtles *Chrysemys picta bellii* and *Pelodiscus sinensis* [17,18], and overlaps the American alligator (*Alligator mississippiensis*) [72], the green anole (*Anolis carolinensis*) [73], and the chicken (*Gallus gallus*) [70].

Statistics for candidate assemblies are found in the Supporting Information online (S2 and S3 Tables). Assembling with SOAPdenovo2 and a user-set k-mer value of 47 results in a contig N50 of 42.7 kbp, a scaffold N50 of 169.2 kbp, with placing 86.9% of contigs on scaffolds, 43.19% GC content, and 1.51% consisting of Ns after gap-closing, which we select for further analysis. RNA-scaffolding vastly improves the contiguity of the initial assembly by increasing the scaffold N50 to 251.6 kbp and with placing 88.7% of contigs on scaffolds, and 1.55% consisting of Ns (Fig 1B). The total length of the draft assembly is very close to our k-mer based estimate of genome size at 2.4 Gbp, which is longer than 1.2 Gbp for the chicken [70] but comparable to 2.6 Gbp for *C*. *picta bellii* [18], 2.2 Gbp for *P*. *sinensis*, and 2.2 Gbp for *C*. *mydas* [17]. We detect 235 out of 248 (94.8%) core eukaryotic genes (CEGs) in the final assembly in at least partial status, and 194 (78.2%) CEGs are complete. We identify 92% of the 3,023 vertebrate-specific BUSCOs in the assembly, including 2,324 (77%) complete and 459 (15%) fragmented (S4 Table). Table 3 summarizes the final assembly statistics. The distribution of GC content across the assembly is similar to that of other published turtle genomes, and differs from more phylogenetically distant reptiles (Fig 2).

**Table 3:** Assembly statistics for the genome of *Gopherus agassizii*.

	Contigs	Scaffolds
N50	42,722 bp	251,635 bp
L50 (Number)	15,759	2,592
Longest	436,052 bp	2,046,553 bp
Total Length	2,363,296,958 bp	2,399,952,228 bp
Number ≥ 1 kbp	106,825	42,911
Number ≥ 100 kbp	2,672	6,264
Proportion gaps	0%	1.55%

### Genome annotation

To maximize the representation of annotated genes, we executed deep transcriptome sequencing of tissues with divergent expression profiles and developmental origins: brain (ectodermal origin), skeletal muscle (mesodermal origin), and lung (endodermal origin). RNA sequencing (NCBI BioProject PRJNA352725) produced 93,284,795 Illumina reads from brain, 107,105,716 reads from lung, and 86,205,948 reads from skeletal muscle, all of which assemble in a transcriptome containing 245,326 contigs including 2,642 (87%) complete and 143 (4.7%) fragmented vertebrate BUSCOs (S4 Table). Genome annotation also uses published data from blood transcriptomes of *G*. *agassizii* (NCBI BioProject PRJNA281763) [11] and publicly available protein sequences. The annotation pipeline identifies 20,172 protein-coding genes (including 85% of vertebrate BUSCOs, S4 Table), 17,173 5’ untranslated regions (UTRs), and 20,417 3’UTRs.

The number of protein-coding genes in the *G*. *agassizii* assembly (20,172) is comparable to the number of predicted genes in other published reptilian genomes, including 21,796 in the western painted turtle [18], 19,327 in the Chinese softshell turtle [17], 19,633 in the green sea turtle [17], 22,962 in the green anole [73], and 15,508 in the chicken [70]. The number of shared one-to-one gene orthologs between Agassiz’s desert tortoise and other reptiles include 15,220 with western painted turtle (S1 Dataset), 12,505 with Chinese softshell turtle (S2 Dataset), 11,491 with chicken (S3 Dataset), and 11,983 with green anole (S4 Dataset). The number of shared one-to-one gene orthologs between Agassiz’s desert tortoise and other reptiles is comparable to the number shared with the human genome (13,284, S5 Dataset). Predicted orthology groups identified by the OrthoFinder analysis shared across human, turtles, archosaurs, and lepidosaurs are made available to the comparative genomics community (S6 Dataset).

**Fig 3:**
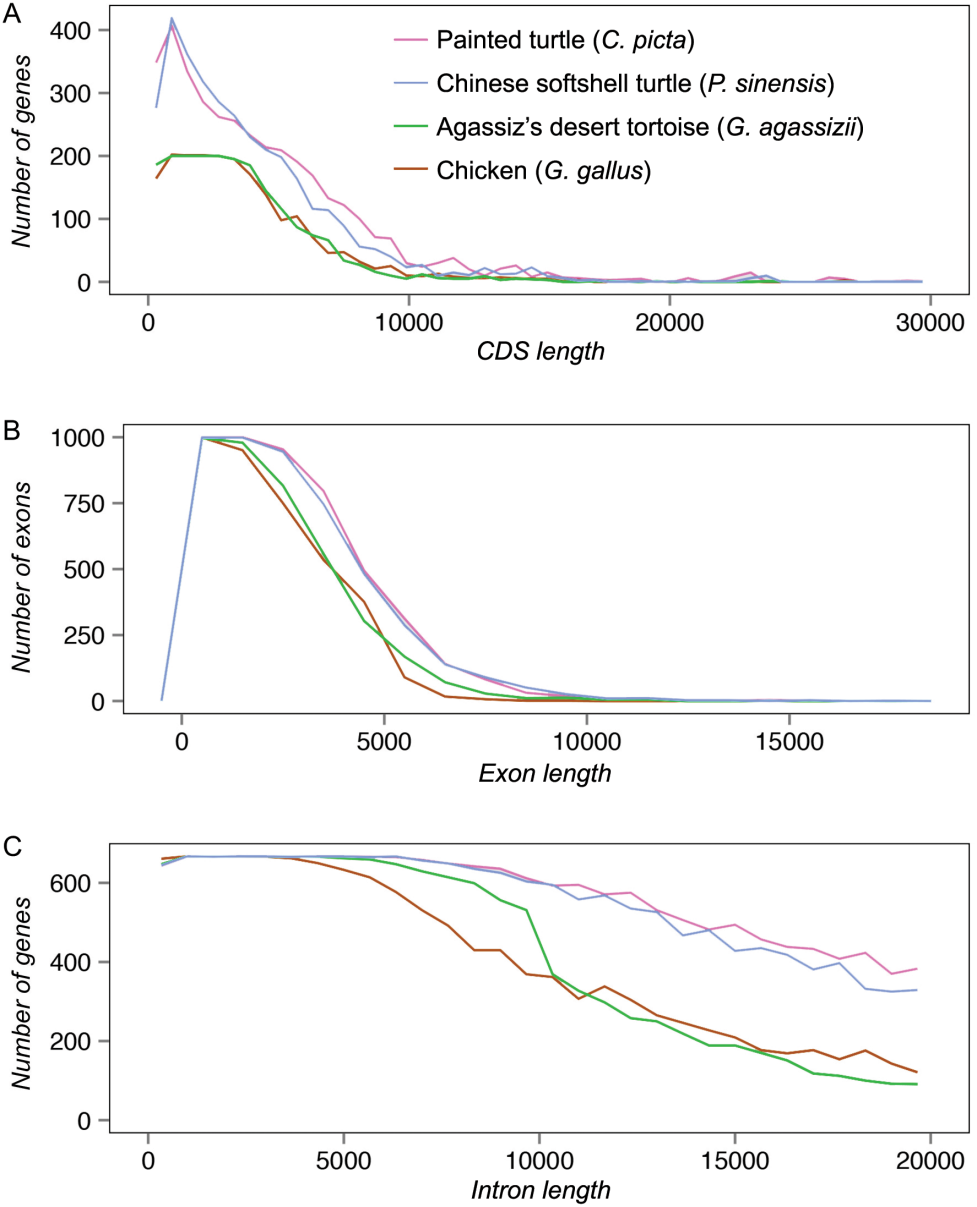
Gene structure of *Gopherus agassizii* compared to other reptiles. Frequency distribution of genes or exons relative to (A) coding DNA sequence (CDS) lengths, (B) exon length and (C) intron length of Agassiz’s desert tortoise, chicken [74], western painted turtle [18], and Chinese softshell turtle [17]. CDS is for genes less than 10 kb.

Gene structure parameters for the *G*. *agassizii* genome build are comparable with that of other reptile genomes, with Agassiz’s desert tortoise being more similar to the chicken than to the two aquatic chelonians (Fig 3). The total coding DNA sequence (CDS) length for Agassiz’s desert tortoise is similar to that of the chicken, but it has fewer genes below the 10 kb CDS length than do the western painted turtle and Chinese softshell turtle (Fig 3A). The frequency distribution of exon length is similar among the four species (Fig 3B) but intron length is most similar for Agassiz’s desert tortoise and chicken; both have fewer large intron genes compared to the other chelonians (Fig 3C). The low number of large introns in Agassiz’s desert tortoise and chicken may be the result of a reduction in intron size in the common ancestor of chelonians and archosaurs [74,75] followed by lineage-specific increases in intron length in western painted and Chinese softshell turtles [76], or may result from independent changes in Agassiz's desert tortoise. The future availability of additional chelonian and archosaurian genomes will help to address this question.

### Repeat analysis

Repeat identification of the *G*. *agassizii* genome using known RepBase elements results in 31% of the genome masked (Table 4). Additional *d*e *novo* repeat identification with RepeatModeler improves the annotations of several classes of repeats, resulting in 43% of the genome being assigned to repeats. These include a diverse array of transposable elements, which comprise 33% of the genome, including DNA transposons, long terminal repeat (LTR) elements, long interspersed nuclear elements (LINEs), and short interspersed elements (SINEs). The genomic proportion of repeats in Agassiz’s desert tortoise is greater than that for the western painted turtle (29%). Slight differences in the abundance of repeat classes exist between the analyzed chelonians, although relative abundances are similar except for DNA transposons, which are more abundant in Agassiz’s desert tortoise (12.4% versus 9.8% in the western painted turtle), as are LTR retrotransposons (7.7% versus 5.2%, respectively). The genomes of both Agassiz’s desert tortoise and the western painted turtle possess a large proportion of CR1 subfamilies at ≤ 5% K2P divergence from their consensus sequence (S1 Fig), suggesting they are recent inserts. Therefore, these elements comprise the most active and common retrotransposons in both chelonians and archosaurs [72,77]. The high percentage (8%) of unclassified interspersed repeats identified by RepeatModeler in the Agassiz’s desert tortoise’s genome, for which we could not find matches in either RepBase or GenBank, suggests that novel elements exist in the genomes of chelonians.

**Table 4:** Repetitive content of the Agassiz’s desert tortoise (*Gopherus agassizii*) and the western painted turtle (*Chrysemys picta bellii*) genomes, estimated with libraries of known repeats (RepBase) and *de novo* repeat identification (RepeatModeler).

	*Gopherus agassizii* (RepBase)		*Gopherus agassizii* (RepBase + RepeatModeler)		*Chrysemys picta bellii* (RepBase)	
Repeat Type^1^	Length (bp)	% genome	Length (bp)	% genome	Length (bp)	% genome
SINEs	42,775,342	1.78	44,092,705	1.84	44,277,662	1.87
LINEs	263,416,736	10.98	276,159,275	11.51	248,848,977	10.52
LTR	165,658,037	6.90	184,823,905	7.70	123,030,173	5.20
DNA Transposons	244,173,277	10.17	297,537,719	12.4	18,952,044	9.80
Unclassified	18,348,096	0.76	203,226,010	8.47	20,076,666	0.80
Small RNA	8,799,494	0.37	9,451,148	0.39	8,527,612	0.36
Satellites	519,850	0.02	1,402,596	0.06	267,675	0.01
Simple Repeats	11,038,402	0.46	19,005,379	0.79	11,395,517	0.48
Low Complexity	2,145,620	0.09	1,714,833	0.07	2,244,030	0.09

^1^SINE, short interspersed nuclear element; LINE, long interspersed nuclear element; LTR, long terminal repeat retrotransposons.

### Slow DNA substitution rates in chelonians

**Fig 4:**
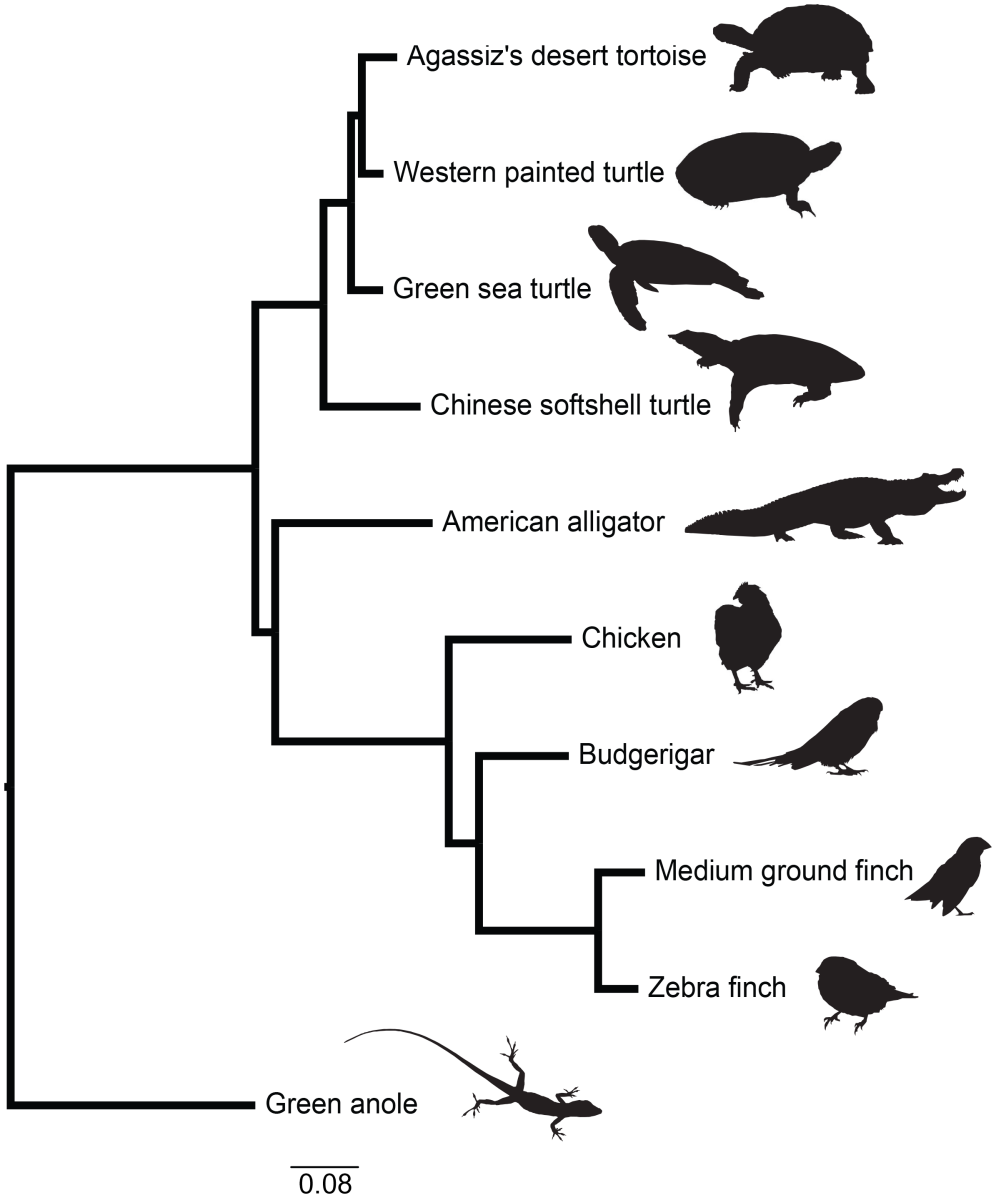
Phylogeny of 10 sauropsids including *Gopherus agassizii*. Phylogeny shows the relationships of sauropsids, including four chelonians, with complete genome sequences. Branch lengths are given in terms of DNA substitutions per site derived from fourfold degenerate sites.

The filtered whole genome alignment (WGA) is just over 217 Mbp in length, including 15% gaps, with an average of 9.23 species represented in each alignment block. The RAxML analysis included full statistical support for the *a priori* expected relationships, including the monophyly of *C*. *picta bellii* and *G*. *agassizii*, *P*. *sinensis* as the outgroup to all other included chelonians, and a monophyletic archosauria. When analyzing 1,815,350 4D sites, branch lengths in terms of substitutions per site on the sauropsid phylogeny (Fig 4, S2 Fig) suggest limited neutral divergence since the most recent common ancestor of chelonians. In contrast, the avian clade has longer internal branches, consistent with faster evolutionary rates in birds compared to other archosaurs [72,77]. Incorporating different divergence times between *Gopherus* and *Chrysemys* (S5 Table) affects the absolute estimated rates but not relative results. These results are similar to previous findings that chelonians have accumulated fewer DNA substitutions over time than other sauropsid lineages [17,18] (S3 Fig, S7 Dataset). This may be due to the unusually long generation times of many chelonians, which correlate with lower substitution rates in reptiles [78].

### Comparative genomics of turtles

The *G*. *agassizii* genome alignments to western painted turtle, green sea turtle, Chinese softshell turtle and chicken are 1.9 Gbp, 1.8 Gbp, 1.3 Gbp, and 0.44 Gbp in length, respectively. There are 223 genes with Ka/Ks > 1 in the tortoise-western painted turtle comparison, and 75, 31, and 23 in the comparisons with green sea turtle, Chinese softshell turtle and chicken, respectively (S8 Dataset). This results in 58, 29, 6, and 23 accelerated genes with clear orthology in the respective comparisons (S9 Dataset), though none passed the p-value threshold for positive selection after the conservative Bonferroni correction for multiple hypothesis testing. Notwithstanding, we report results of all genes with Ka/Ks > 1, because both positive selection and relaxed functional constraint can affect the evolution of phenotypes [79].

**Fig 5:**
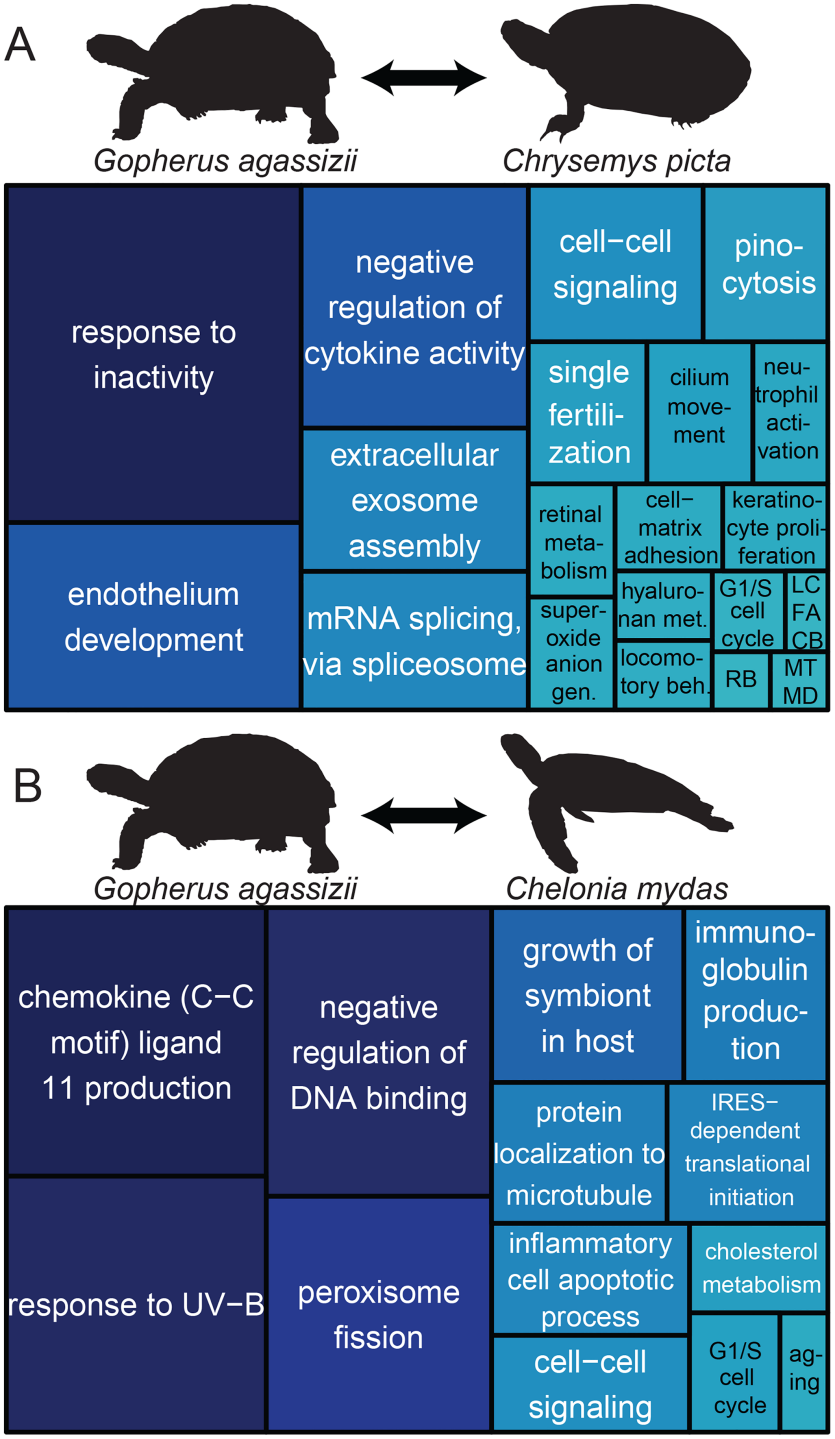
Comparative genomics of chelonians. Representation of Gene Ontology terms for biological processes shared by genes with pairwise Ka/Ks > 1 in comparisons of *Gopherus agassizii* to (A) *Chrysemys picta bellii* and (B) *Chelonia mydas*. Treemap boxes are both sized and colored by uniqueness. Abbreviations in (A): RB, receptor biosynthesis, LCFACB, long chain fatty acyl-CoA biosynthesis, MTMD, membrane to membrane docking.

The western painted turtle is the taxonomically closest genome-enabled species to Agassiz’s desert tortoise, and as a temperate aquatic species its comparison with desert tortoise can shed light on the evolution of many desert tortoise traits. Indeed, the desert tortoise-painted turtle comparison yielded the highest number of genes with Ka/Ks > 1. Many of the 58 accelerated genes may be relevant to tortoise adaptations (Fig 5A), such as *CD34*. This gene codes for an adhesion molecule on circulating cells that contribute to vascular repair following glomerular injury [80]. Optimal renal function is critical for organisms living in the desert, such as *G*. *agassizii*. *CSTA* and *SDR16C5* may also link to important chelonian adaptations, as they are involved in keratinocyte differentiation (GO:0030216) and proliferation (GO:0043616), respectively. This implies involvement in the formation of reptilian scales and feathers including the chelonian shell [81]. Other accelerated genes control responses to oxidative stress, including *VIMP* and *SOD1*. This is consistent with the initial analysis of the western painted turtle genome, which showed molecular evidence for adaptations for anoxic conditions [18]. Accelerated genes between the Agassiz’s desert tortoise and western painted turtle involve function in immunity, including *SOD1*, *FFAR4*, *CXCL11*, CXCL8, *CD4*, *IL10*, and *NFAM1*. These genes regulate cytokine activity. The comparison with green sea turtle (Fig 5B) also yields amino acid changes in genes involved in immune function such as *TNFSF4* and *IFNG*, both of which regulate CD4^+^ T cell/antigen presenting cell interactions, and *PGLYRP1* which is a peptidoglycan-recognizing protein involved in response to bacterial infection. Agassiz’s desert tortoise and the green sea turtle share several accelerated genes involved in apoptosis and aging, including *BCL2A1* and *GZMA*.

Analyses fail to detect many genes with Ka/Ks > 1 in the comparison of Agassiz’s desert tortoise and the Chinese softshell turtle, yet *CD4* is also accelerated as in the comparison with the western painted turtle. Also, as in the green sea turtle comparison, the chicken comparison shows accelerated evolution in *TNFSF4*. Given their phylogenetic relationships, these changes to the adaptive immune system appear unique to Agassiz’s desert tortoise within these taxa. We also find amino acid differences between chicken and desert tortoise in genes such as *MPV17*, which is involved in mitochondrial homeostasis, suggesting different adaptive pathways controlling metabolism in endotherms (chicken) and ectotherms (tortoise). Finally, *AQP10* is a potentially important accelerated gene from the chicken comparison, which functions as a water-selective channel that can permeate neutral solutes such as glycerol and urea [82]. Altogether, these analyses of protein coding genes suggest that chelonian adaptations relate to shell development and anoxia, and tortoise-specific adaptations involve longevity, osmoregulation and water balance, immune function, and metabolism. Such results provide a foundation for future chelonian studies in conservation and organismal function.

### Comparative genomics of the desert tortoise species complex

After filtering for quality and coverage, *G*. *agassizii*, *G*. *morafkai* and *G*. *evgoodei* have 10,086, 2,593 and 23,261 fixed unique SNPs, respectively. Of these variants, the numbers occurring in genes with assigned orthology to *Anolis carolinensis* are 910, 360, and 1,340, respectively. Analyses using g:GOSt identify 59 (BP: 27, CC: 28, MF: 4), 23 (BP: 1, CC: 20, MF: 2), and 95 (BP: 42, CC: 39, MF: 14) enriched GO categories, respectively. The conversion of genes to GO terms in g:Convert produces 8,928 GO terms for *G*. *agassizii*, 3,590 for *G*. *morafkai*, and 13,455 for *G*. *evgoodei*, including duplicates by different genes yielding the same GO term (S6–S8 Tables, S5 Fig). The GO categories most enriched for *G*. *agassizii* (Table 5) are: RNA metabolism and processing (RNA processing GO:0006396, mRNA metabolic process GO:0016071, mRNA processing GO:0006397); alteration of polypeptides, polynucleotides, polysaccharides, or other biological macromolecule (macromolecule modification GO:0043412, cellular macromolecule metabolic process GO:0044260, cellular protein modification process GO:0006464); and other basic metabolic processes (primary metabolic process GO:0044238, heterocycle metabolic process GO:0046483, cellular metabolic process GO:0044237, nucleobase-containing compound metabolic process GO:0006139, cellular nitrogen compound metabolic process GO:0034641, organic substance metabolic process GO:0071704, cellular aromatic compound metabolic process GO:0006725).

**Table 5:** Gene Ontology categories most enriched for fixed unique SNPs in *G*. *agassizii* compared with *G*. *morafkai* and *G*. *evgoodei*. These results are for Biological Processes, for a complete listing see S6–S8 Tables.

Gene Ontology (GO) category	GO ID	Number of enriched genes	p-value
RNA processing	GO:0006396	59	1.23E-07
macromolecule modification	GO:0043412	193	2.66E-04
cellular macromolecule metabolic process	GO:0044260	371	4.43E-04
primary metabolic process	GO:0044238	446	9.94E-04
cellular protein metabolic process	GO:0044267	226	1.07E-03
cellular nitrogen compound metabolic process	GO:0034641	280	1.59E-03
cellular metabolic process	GO:0044237	433	2.34E-03
protein modification process	GO:0036211	182	2.55E-03
cellular protein modification process	GO:0006464	182	2.55E-03
heterocycle metabolic process	GO:0046483	258	2.90E-03
nucleobase-containing compound metabolic process	GO:0006139	253	3.21E-03
macromolecule metabolic process	GO:0043170	393	4.47E-03
mRNA metabolic process	GO:0016071	32	6.38E-03
organic substance metabolic process	GO:0071704	454	6.46E-03
chromatin modification	GO:0016568	42	6.96E-03
cellular aromatic compound metabolic process	GO:0006725	256	7.53E-03

Basic cellular processes are necessary for the higher functions *G*. *agassizii* uses to survive the environmentally harsh Mojave Desert, including circadian rhythm, aging, response to UV, regulation of urine volume, and humoral and innate immune responses (Table 6). The enriched GO category of macromolecule modification (GO:0043412) includes genes involved in such biological processes that may be important to the adaptation of *G*. *agassizii* to its environment (S6–S8 Tables). *CRY1* (Cryptochrome-1), and *CSNK1D* (Casein kinase I isoform delta) are also involved with circadian rhythm (GO:0007623). In the northern house mosquito, functional *CRY1* is required for overwintering diapause or dormancy [83], although the role of circadian genes in regulation of brumation remains to be determined. Five genes overlap with response to UV (GO:0009411): *ERCC6* (DNA excision repair protein ERCC-6), *HYAL2* (Hyaluronidase-2), *PPID* (Peptidyl-prolyl cis-trans isomerase D), *UBE2B* (Ubiquitin-conjugating enzyme E2 B), and USP28 (Ubiquitin carboxyl-terminal hydrolase 28). *HYAL2* overlaps with other genes in the category for regulation of urine volume (GO:0035809). With an estimated lifespan of up to 53 years in the wild [4], Agassiz’s desert tortoise allows for investigations into the genetic regulation of aging, making it a potentially important species for the understanding of many human diseases [15,16]. Six genes overlap with aging (GO: 0007568): *ABL1* (Tyrosine-protein kinase ABL1), *CARM1* (Histone-arginine methyltransferase CARM1), *HYAL2*, *PRKCD* (Protein kinase C delta type), *RPN2* (Dolichyl-diphosphooligosaccharide-protein glycosyltransferase subunit 2), and *TGFB1* (Transforming growth factor beta-1). These genes with functional enrichment across *Gopherus* spp. and which overlap with relevant desert-related processes warrant further investigation. Indeed the role that such macromolecular modification genes play in desert adaptation is a question with broad relevance, and may be important to understanding the biology of this threatened species.

**Table 6:** Overlapping genes in the two most enriched GO categories for fixed unique SNPs in *G*. *agassizii* compared with key functional categories. No overlap was identified with GO:0002456 T cell mediated immunity.

*G*. *agassizii* GO category	Overlapping GO category	Gene symbol	Description
GO:0006396 RNA processing	GO:0045087 innate immune response	SRPK1	Serine and arginine rich splicing factors protein kinase 1
GO:0043412 macromolecule modification	GO: 0007568 aging	ABL1	Tyrosine-protein kinase ABL1
		CARM1	Histone-arginine methyltransferase CARM1
		HYAL2	Hyaluronidase-2
		PRKCD	Protein kinase C delta type
		RPN2	Dolichyl-diphosphooligosaccharide—protein glycosyltransferase subunit 2
		TGFB1	Transforming growth factor beta-1
	GO:0007623 circadian rhythm	CRY1	Cryptochrome-1
		CSNK1D	Casein kinase I isoform delta
	GO:0009411 response to UV	ERCC6	DNA excision repair protein ERCC-6
		HYAL2	Hyaluronidase-2
		PPID	Peptidyl-prolyl cis-trans isomerase D
		UBE2B	Ubiquitin-conjugating enzyme E2 B
		USP28	Ubiquitin carboxyl-terminal hydrolase 28
	GO:0019724 B cell mediated immunity	PAXIP1	PAX-interacting protein 1
		PRKCD	Protein kinase C delta type
		TGFB1	Transforming growth factor beta-1
	GO:0045087 innate immune response	ABL1	Tyrosine-protein kinase ABL1
		CDC37	Hsp90 co-chaperone Cdc37
		IFIH1	Interferon-induced helicase C domain-containing protein 1
		NOD1	Nucleotide-binding oligomerization domain-containing protein 1
		PELI1	E3 ubiquitin-protein ligase pellino homolog 1
		PRKCD	Protein kinase C delta type
		SRPK1	SRSF protein kinase 1
		TAB1	TGF-beta-activated kinase 1 and MAP3K7-binding protein 1
		TGFB1	Transforming growth factor beta-1
	GO:0035809 regulation of urine volume	HYAL2	Hyaluronidase-2

Immune response is important for *G*. *agassizii* due to the respiratory tract disease that may be driving population declines (Table 6). No genes with fixed variants overlap with T cell mediated immunity (GO:0002456), yet three genes overlap with humoral/B cell mediated immunity (GO:0019724): *PAXIP1* (PAX-interacting protein 1), *PRKC* (Protein kinase C delta type), and *TGFB1*. Nine genes overlap with innate immune response (GO:0045087): *ABL1*, *CDC37* (Hsp90 co-chaperone Cdc37), *IFIH1* (Interferon-induced helicase C domain-containing protein 1), *NOD1* (Nucleotide-binding oligomerization domain-containing protein 1), *PELI1* (E3 ubiquitin-protein ligase pellino homolog 1), *PRKCD* (Protein kinase C delta type), *SRPK1* (Serine and arginine rich splicing factors protein kinase 1), *TAB1* (TGF-beta-activated kinase 1 and MAP3K7-binding protein 1), and *TGFB1*. Only *SRPK1* overlaps with the RNA processing group (GO:0006396) and the more functionally focused groups for innate immune response (GO:0045087). Given the susceptibility of *G*. *agassizii* to upper respiratory disease, variants that would influence the immune response are of high interest to understand host factors involved in pathogenesis. Genes with fixed differences between species of the *Gopherus* desert tortoise species complex, as well as genes found to be under accelerated evolution across chelonians, are summarized in Table 7.

**Table 7:** Genes under accelerated evolution across chelonians and genes with fixed differences between species in the *Gopherus* desert tortoise species complex according to this study.

Gene	Gene symbol	Locus*
**Genes under accelerated evolution across chelonians**		
Hematopoietic progenitor cell antigen	*CD34*	gopAga1_00002251
Cystatin-A	*CSTA*	scaffold10781 (XM_005291323)
Short Chain Dehydrogenase/Reductase Family 16c Member 5	*SDR16C5*	gopAga1_00014840
Selenoprotein S	*VIMP*	gopAga1_00004579
Superoxide dismutase 1	*SOD1*	scaffold518 (XM_005283906)
Free fatty acid receptor 4	*FFAR4*	gopAga1_00009948
C-x-c motif chemokine ligand 11	*CXCL11*	gopAga1_00011528
Interleukin 10	*IL10*	gopAga1_00002900
Nfat activating protein with itam motif 1	*NFAM1*	gopAga1_00016407
Tumor necrosis factor superfamily member 4	*TNFSF4*	scaffold21701 (XM_007061249)
Interferon gamma	*IFNG*	gopAga1_00016782
Peptidoglycan recognition protein 1	*PGLYRP1*	gopAga1_00017264
Bcl2 related protein a1	*BCL2A1*	gopAga1_00011769
Granzyme A	*GZMA*	gopAga1_00003655
**Genes with fixed differences in the *Gopherus* species complex**		
Cryptochrome-1	*CRY1*	gopAga1_00014628
Casein kinase i isoform delta	*CSNK1D *	gopAga1_00008116
DNA excision repair protein ercc-6	*ERCC6*	gopAga1_00012364
Hyaluronidase-2	*HYAL2*	gopAga1_00019510
Peptidyl-prolyl cis-trans isomerase d	*PPID *	gopAga1_00006436
Ubiquitin-conjugating enzyme e2 b	*UBE2B*	gopAga1_00015001
Ubiquitin carboxyl-terminal hydrolase 28	*USP28 *	gopAga1_00008255
Tyrosine-protein kinase abl1	*ABL1*	gopAga1_00009847
Histone-arginine 8 methyltransferase carm1	*CARM1*	gopAga1_00007934
Protein kinase c delta type	*PRKCD*	gopAga1_00004289
Dolichyl-diphosphooligosaccharide-protein glycosyltransferase subunit 2	*RPN2*	gopAga1_00010670
Transforming growth factor beta-1	*TGFB1*	gopAga1_00017303
Casein kinase I isoform delta	*CSNK1D *	gopAga1_00008116

*Identification number of annotated *Gopherus agassizii* protein, otherwise *G*. *agassizii* scaffold and RefSeq accession number of mapped locus from LASTZ (see Methods).

## Conclusions

Agassiz’s desert tortoise (*Gopherus agassizii*) is listed as a threatened species. Here, we report a *de novo* genome assembly and annotation for *G*. *agassizii* to serve as a conservation resource for assessing genetic diversity across the species’ range, identifying genes involved in adaptation to warm deserts, and genomic responses to the respiratory disease that is threatening desert tortoise populations. We generated a draft genome assembly and annotation based on deep transcriptome sequencing of four tissues from adult tortoises. The genome assembly represents 2.4 Gbp with a scaffold N50 length of 251.6 kbp, and a 43% genomic proportion of repetitive elements. Genome annotation identifies 20,172 protein-coding genes, including an estimated 95% of core eukaryotic genes, and is comparable to predicted gene numbers in turtles. We demonstrate that this draft genome will be a useful reference for the future mapping of markers in genetic studies of the evolution of *G*. *agassizii*, such as defining the boundaries of the hybrid zone with Morafka’s desert tortoise (*G*. *morafkai*). Our analyses of gene sequences lay the groundwork for understanding the genetic basis of adaptations to arid environments for this species and its congeners, and potentially other desert-adapted species, including identifying genes involved in response to UV and regulation of urine volume. They may also advance our understanding of characteristics of interest to humans, such as longevity, and management of diseases that plague this species and other chelonians.

## Supporting information

S1 Fig Divergence summary of repetitive elements in the genome of *Gopherus agassizii*. Distribution of repetitive elements in the *G*. *agassizii* genome including DNA transposons, long terminal repeat (LTR) elements, long interspersed nuclear elements (LINEs), short interspersed elements (SINEs), and unknown elements.
(TIFF)

S2 Fig Phylogeny of sauropsids with sequenced genomes (see Fig 4). Branch lengths are in terms of substitutions per site derived from fourfold degenerate sites. Nodes are labeled in reference with S5 Table.
(TIFF)

S3 Fig DNA substitution rates in sauropsids derived from divergence at fourfold degenerate sites in two node constraint scenarios (see S5 Table). (A) Estimated divergence of *Gopherus agassizii* and *Chrysemys picta bellii* without constraint. (B) Fixed age of node 9 (see S2 Fig.) at 74.2 million years.
(TIFF)

S4 Fig Representation of gene ontology terms for biological processes shared by genes with Ka/Ks > 1 in the pairwise comparison of *Gopherus agassizii* and *Gallus gallus*. Treemap boxes are sized according to uniqueness.
(TIFF)

S5 Fig Speciation genomics of desert tortoises. (A) Phylogenetic relationships and divergence times of three desert tortoise species. Total RNA was sequenced for three individuals per taxon and mapped to the *Gopherus agassizii* assembly. (B) Representation of Gene Ontology terms shared by genes with fixed unique single nucleotide polymorphisms in each species of desert tortoise. Treemap boxes are sized according to uniqueness.
(TIFF)

S1 Table Data on tissue used for (A) DNA and (B) RNA sequencing. (XLS)

S2 Table Statistics for candidate single-end assemblies (“unitigs”) generated using different user-set k-mer values with ABySS. The unitig with the largest N50 (k-mer = 83) was chosen for further assembly using ABySS.
(XLSX)

S3 Table Statistics for candidate assemblies using different assemblers, methods and user-set k-mer values. (XLSX)

S4 Table Results from the Benchmarking Universal Single Copy Orthologs (BUSCO) analyses of the final *Gopherus agassizii* genome assembly, transcriptome assembly, and gene set and comparison with other genomic datasets. (XLSX)

S5 Table Node constraints and estimates for joint divergence time (in millions of years) and substitution rate analyses. Node labels are from S2 Fig.
(XLSX)

S6 Table Enriched GO categories using unique, fixed variants per taxon for *Gopherus agassizii*. (XLSX)

S7 Table Enriched GO categories using unique, fixed variants per taxon for *Gopherus morafkai*. (XLSX)

S8 Table Enriched GO categories using unique, fixed variants per taxon for *Gopherus evgoodei*. (XLSX)

S1 Appendix Supporting methods. (A) Commands used for the various steps in the genome assembly process. (B) Commands used to filter the variant file (.vcf) from FreeBayes using SnpSft, and to intersect it with the *Gopherus agassizii* annotation using bedtools.
(DOCX)

S1 Dataset Assigned 1:1 orthologs between Agassiz’s desert tortoise (*Gopherus agassizii*) and western painted turtle (*Chrysemys picta bellii*). (XLS)

S2 Dataset Assigned 1:1 orthologs between Agassiz’s desert tortoise (*Gopherus agassizii)* and Chinese softshell turtle (*Pelodiscus sinensis*). (XLS)

S3 Dataset Assigned 1:1 orthologs between Agassiz’s desert tortoise (*Gopherus agassizii)* and chicken (*Gallus gallus*). (XLS)

S4 Dataset Assigned 1:1 orthologs between Agassiz’s desert tortoise (*Gopherus agassizii)* and green anole (*Anolis carolinensis*). (XLS)

S5 Dataset Assigned 1:1 orthologs between Agassiz’s desert tortoise (*Gopherus agassizii*) and human (*Homo sapiens*). (XLS)

S6 Dataset Ortholog groups shared between Agassiz’s desert tortoise (*Gopherus agassizii*), western painted turtle (*Chrysemys picta bellii*), Chinese softshell turtle (*Pelodiscus sinensis*), chicken (*Gallus gallus*), alligator (*Alligator mississippiensis*), green anole (*Anolis carolinensis*) and human (*Homo sapiens*). (CSV)

S7 Dataset Estimated DNA substitution rate analysis and results using penalized likelihood. (ZIP)

S8 Dataset Output from Ka/Ks analysis for all genes compared between Agassiz’s desert tortoise (*Gopherus agassizii*) and western painted turtle (*Chrysemys picta bellii*), Chinese softshell turtle (*Pelodiscus sinensis*), green sea turtle (*Chelonia mydas*), and chicken (*Gallus gallus*). (ZIP)

S9 Dataset Biomart results for human Ensembl gene ID and Gene Ontology categories for accelerated genes with Ka/Ks > 1 between Agassiz’s desert tortoise (*Gopherus agassizii*) and western painted turtle (*Chrysemys picta bellii*), Chinese softshell turtle (*Pelodiscus sinensis*), green sea turtle (*Chelonia mydas*), and chicken (*Gallus gallus*). (XLS)
