# Supplementary figures and images for: The Agassiz’s desert tortoise genome provides a resource for the conservation of a threatened species
Source: PLoS One. 2017 May 31;12(5):e0177708. doi: 10.1371/journal.pone.0177708 (PMC5451010; doi:10.1371/journal.pone.0177708)

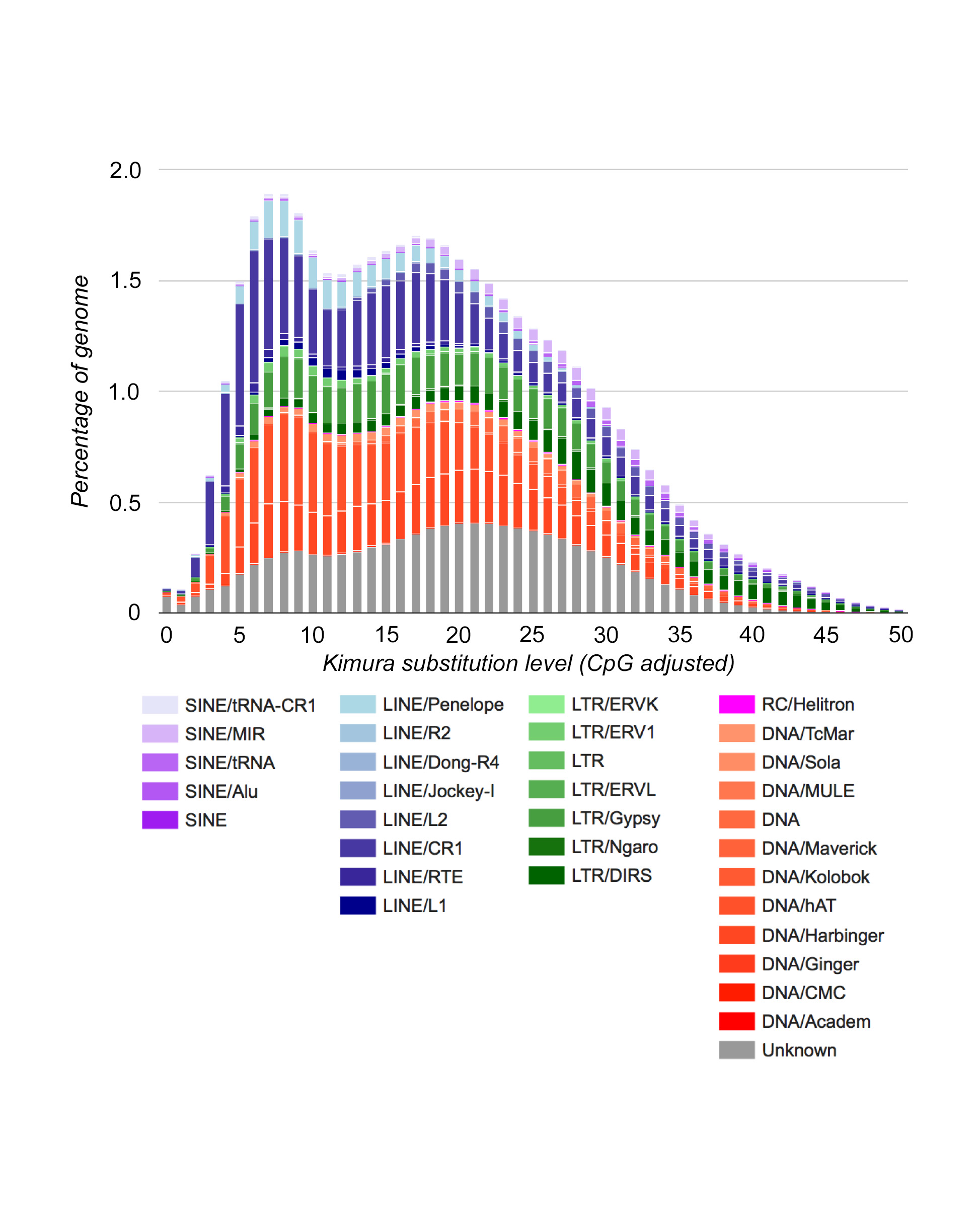

Supplement: S1 Fig — Distribution of repetitive elements in the G. agassizii genome including DNA transposons, long terminal repeat (LTR) elements, long interspersed nuclear elements (LINEs), short interspersed elements (SINEs), and unknown elements. (TIFF) [file pone.0177708.s001.tiff]

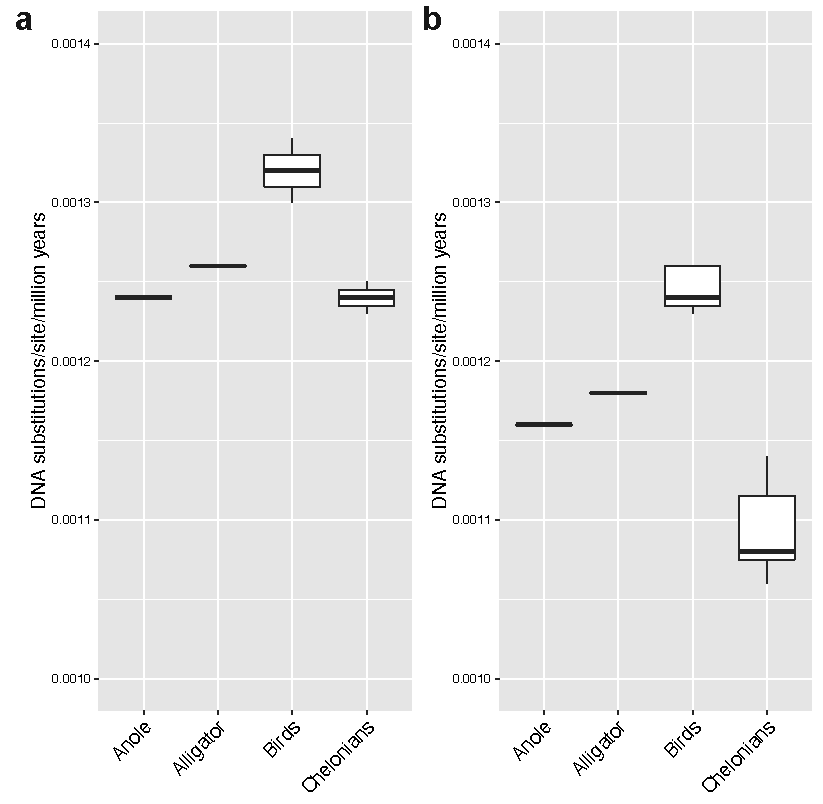

Supplement: S3 Fig — (A) Estimated divergence of Gopherus agassizii and Chrysemys picta bellii without constraint. (B) Fixed age of node 9 (see S2 Fig.) at 74.2 million years. (TIFF) [file pone.0177708.s003.tiff]

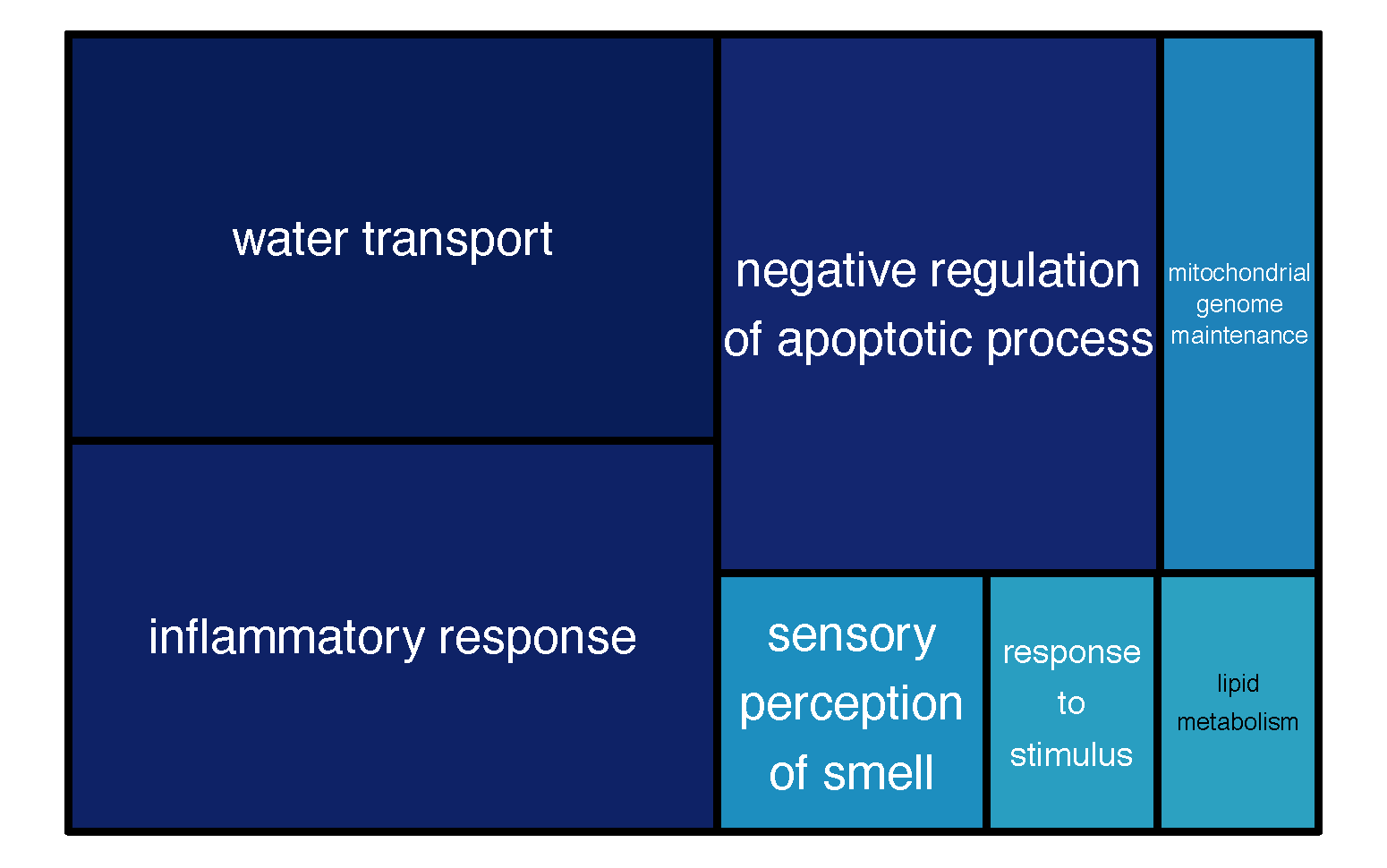

Supplement: S4 Fig — Treemap boxes are sized according to uniqueness. (TIFF) [file pone.0177708.s004.tiff]

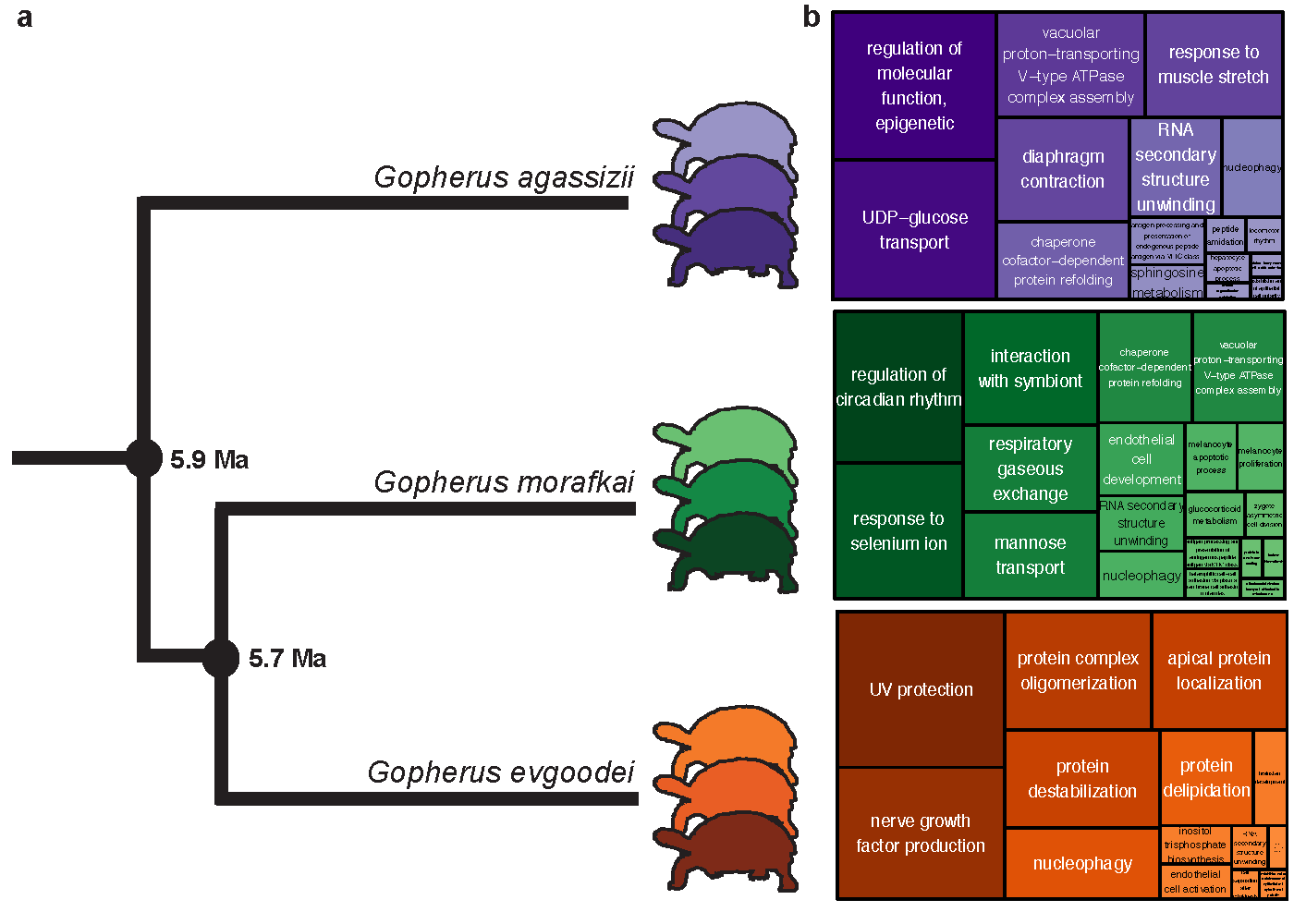

Supplement: S5 Fig — (A) Phylogenetic relationships and divergence times of three desert tortoise species. Total RNA was sequenced for three individuals per taxon and mapped to the Gopherus agassizii assembly. (B) Representation of Gene Ontology terms shared by genes with fixed unique single nucleotide polymorphisms in each species of desert tortoise. Treemap boxes are sized according to uniqueness. (TIFF) [file pone.0177708.s005.tiff]
