# Supplementary material for: The Agassiz’s desert tortoise genome provides a resource for the conservation of a threatened species
Source: PLoS One. 2017 May 31;12(5):e0177708. doi: 10.1371/journal.pone.0177708 (PMC5451010; doi:10.1371/journal.pone.0177708)
Supplement: S1 Appendix — (A) Commands used for the various steps in the genome assembly process. (B) Commands used to filter the variant file (.vcf) from FreeBayes using SnpSft, and to intersect it with the Gopherus agassizii annotation using bedtools. (DOCX) [file pone.0177708.s014.docx]

A. The following is a summary of the commands used for the various steps in the genome assembly process.

SOAP KmerFreq commands:

KmerFreq_HA -k 27 -l reads_list.txt -p Gag27 -t 32 -L 104 >kmerfreq_all27.cout 2>kmerfreq_all27.cerr

Paired-end error corrector:

Corrector_HA -k 27 -l 3 -t 16 Gag27.freq.gz PE_reads_list.txt >corrGag27_PE.cout 2>corrGag27_PE.cerr

Single-end error corrector:

Corrector_HA -k 27 -j 0 -l 3 -t 16 Gag27.freq.gz SE_reads_list.txt >corrGag27_SE.cout 2>corrGag27_SE.cerr

platanus commands:

platanus assemble -o Gag.platanus -f Gag_200bp_R1.fq Gag_200bp_R2.fq Gag_200bp_single_flash.fq Gag_200bp_single.fq Gag_300bp_R1.fq Gag_300bp_R2.fq Gag_300bp_single.fq Gag_1kb_R1.fq Gag_1kb_R2.fq Gag_1kb_single.fq -s 5 -a 7.0 -d 0.3  -t 16 -m 400 2>ass.log.txt

platanus scaffold -o Gag.platanus.scaff -c Gag.platanus_contig.fa -b Gag.platanus_contigBubble.fa -IP1 Gag_200bp_R1.fq Gag_200bp_R2.fq -IP2 Gag_300bp_R1.fq Gag_300bp_R2.fq -IP3 Gag_1kb_R1.fq Gag_1kb_R2.fq -OP1 Gag.2K.R1.fastq Gag.2K.R2.fastq -OP2 Gag.4K.R1.fastq Gag.4K.R2.fastq -OP3 Gag.10K.R1.fastq Gag.10K.R2.fastq -t 16 2>scaff.log.txt

platanus gap_close -o Gag.platanus.scaf.gapclose -c Gag.platanus.scaff_scaffold.fa -f Gag_200bp_single_flash.fq Gag_200bp_single.fq Gag_300bp_single.fq Gag_1kb_single.fq -IP1 Gag_200bp_R1.fq Gag_200bp_R2.fq -IP2 Gag_300bp_R1.fq Gag_300bp_R2.fq -IP3 Gag_1kb_R1.fq Gag_1kb_R2.fq -OP1 Gag.2K.R1.fastq Gag.2K.R2.fastq -OP2 Gag.4K.R1.fastq Gag.4K.R2.fastq -OP3 Gag.10K.R1.fastq Gag.10K.R2.fastq -t 16 2>gap_close.log.txt

ABySS unitig command (as a PBS array on a cluster):

#!/bin/bash

#PBS -lnodes=6:ppn=12:westmereEP

#PBS -l walltime=96:00:00

cd $PBS_O_WORKDIR

KMER=$PBS_ARRAYID

Gag1kb_R1=/scratch/mtollis/Gopherus/workdir/final_data/Gag_1kb_R1.fq.gz

Gag1kb_R2=/scratch/mtollis/Gopherus/workdir/final_data/Gag_1kb_R2.fq.gz

Gag1kb_S=/scratch/mtollis/Gopherus/workdir/final_data/Gag_1kb_single.fq.gz

Gag200_R1=/scratch/mtollis/Gopherus/workdir/final_data/Gag_200bp_R1.fq.gz

Gag200_R2=/scratch/mtollis/Gopherus/workdir/final_data/Gag_200bp_R2.fq.gz

Gag200_S1=/scratch/mtollis/Gopherus/workdir/final_data/Gag_200bp_single_flash.fq.gz

Gag200_S2=/scratch/mtollis/Gopherus/workdir/final_data/Gag_200bp_single.fq.gz

Gag300_R1=/scratch/mtollis/Gopherus/workdir/final_data/Gag_300bp_R1.fq.gz

Gag300_R2=/scratch/mtollis/Gopherus/workdir/final_data/Gag_300bp_R2.fq.gz

Gag300_S=/scratch/mtollis/Gopherus/workdir/final_data/Gag_300bp_single.fq.gz

module load openmpi/1.7.2-intel-13.0/

mkdir -p /scratch/mtollis/Gopherus/workdir/abyss/unitig/$PBS_ARRAYID

cd /scratch/mtollis/Gopherus/workdir/abyss/unitig/$PBS_ARRAYID

export TMPDIR=/scratch/mtollis/Gopherus/workdir/abyss/tmp

mkdir -p $TMPDIR

mpirun -np 72 ABYSS-P -k$KMER -q3 --coverage-hist=coverage.hist -s Gag_k$PBS_ARRAYID-bubbles.fa -o Gag_k$PBS_ARRAYID-1.fa $Gag1kb_R1 $Gag1kb_R2 $Gag1kb_S $Gag200_R1 $Gag200_R2 $Gag200_S1 $Gag200_S2 $Gag300_R1 $Gag300_R2 $Gag300_S

ABySS contig and scaffold command:

#!/bin/bash

#PBS -lnodes=1:ppn=32:X7560

#PBS -l walltime=96:00:00

cd $PBS_O_WORKDIR

Gag1kb_R1=/scratch/mtollis/Gopherus/workdir/final_data/Gag_1kb_R1.fq.gz

Gag1kb_R2=/scratch/mtollis/Gopherus/workdir/final_data/Gag_1kb_R2.fq.gz

Gag1kb_S=/scratch/mtollis/Gopherus/workdir/final_data/Gag_1kb_single.fq.gz

Gag200_R1=/scratch/mtollis/Gopherus/workdir/final_data/Gag_200bp_R1.fq.gz

Gag200_R2=/scratch/mtollis/Gopherus/workdir/final_data/Gag_200bp_R2.fq.gz

Gag200_S1=/scratch/mtollis/Gopherus/workdir/final_data/Gag_200bp_single_flash.fq.gz

Gag200_S2=/scratch/mtollis/Gopherus/workdir/final_data/Gag_200bp_single.fq.gz

Gag300_R1=/scratch/mtollis/Gopherus/workdir/final_data/Gag_300bp_R1.fq.gz

Gag300_R2=/scratch/mtollis/Gopherus/workdir/final_data/Gag_300bp_R2.fq.gz

Gag300_S=/scratch/mtollis/Gopherus/workdir/final_data/Gag_300bp_single.fq.gz

Gag_2KMP_R1=/scratch/mtollis/Gopherus/matepairs/paired.cat/Gag.2K.R1.fastq.gz

Gag_2KMP_R2=/scratch/mtollis/Gopherus/matepairs/paired.cat/Gag.2K.R2.fastq.gz

Gag_4KMP_R1=/scratch/mtollis/Gopherus/matepairs/paired.cat/Gag.4K.R1.fastq.gz

Gag_4KMP_R2=/scratch/mtollis/Gopherus/matepairs/paired.cat/Gag.4K.R2.fastq.gz

Gag_10KMP_R1=/scratch/mtollis/Gopherus/matepairs/paired.cat/Gag.10K.R1.fastq.gz

Gag_10KMP_R2=/scratch/mtollis/Gopherus/matepairs/paired.cat/Gag.10K.R2.fastq.gz

module load openmpi/1.7.2-intel-13.0/

abyss-pe j=16 np=32 k=83 E=0 s=200 n=3 v=-v l=20 name=Gag_k83 C=/scratch/mtollis/Gopherus/workdir/contig/83 lib='pe1kb pe300 pe200' mp='mp2k mp4k mp10k' mp2k_de=--rf mp4k_de=--rf mp10k_de=--rf \

pe200="$Gag200_R1 $Gag200_R2" pe300="$Gag300_R1 $Gag300_R2" pe1kb="$Gag1kb_R1 $Gag1kb_R2" mp2k="$Gag_2KMP_R1 $Gag_2KMP_R2" mp4k="$Gag_4KMP_R1 $Gag_4KMP_R2" mp10k="$Gag_10KMP_R1 $Gag_10KMP_R2" \

se="$Gag1kb_S $Gag200_S1 $Gag200_S2 $Gag300_S"

SSPACE library file:

lib1 bowtie /media/GONZO/workdir/final_data/Gag_1kb_R1.fq.gz /media/GONZO/workdir/final_data/Gag_1kb_R2.fq.gz 1000 0.5 FR

lib2 bowtie /media/GONZO/Gopherus_matepair_libraries/matepairs/paired.cat/Gag.2K.R1.fastq.gz /media/GONZO/Gopherus_matepair_libraries/matepairs/paired.cat/Gag.2K.R2.fastq.gz 2000 0.95 RF

lib3 bowtie /media/GONZO/Gopherus_matepair_libraries/matepairs/paired.cat/Gag.4K.R1.fastq.gz /media/GONZO/Gopherus_matepair_libraries/matepairs/paired.cat/Gag.4K.R2.fastq.gz 4000 0.95 RF

lib4 bowtie /media/GONZO/Gopherus_matepair_libraries/matepairs/paired.cat/Gag.10K.R1.fastq.gz /media/GONZO/Gopherus_matepair_libraries/matepairs/paired.cat/Gag.10K.R2.fastq.gz 10000 0.95 RF

SSPACE command:

SSPACE_Standard_v3.0.pl -l Gag.library.file.2 -s Gag_k83-contigs.fa -x 0 -o 15 -m 85 -a 0.5 -z 200 -T 24 -b Gag.SSPACE.2

SOAPdenovo2 config file:

#maximal read length

max_rd_len=200

[LIB]

avg_ins=189

reverse_seq=0

asm_flags=3

pair_num_cutoff=3

map_len=32

rank=1

q1=/scratch/mtollis/Gopherus/workdir/final_data/Gag_200bp_R1.fq

q2=/scratch/mtollis/Gopherus/workdir/final_data/Gag_200bp_R2.fq

q=/scratch/mtollis/Gopherus/workdir/final_data/Gag_200bp_single_flash.fq

q=/scratch/mtollis/Gopherus/workdir/final_data/Gag_200bp_single.fq

[LIB]

avg_ins=265

reverse_seq=0

asm_flags=3

pair_num_cutoff=3

map_len=32

rank=2

q1=/scratch/mtollis/Gopherus/workdir/final_data/Gag_300bp_R1.fq

q2=/scratch/mtollis/Gopherus/workdir/final_data/Gag_300bp_R2.fq

q=/scratch/mtollis/Gopherus/workdir/final_data/Gag_300bp_single.fq

[LIB]

avg_ins=788

reverse_seq=0

asm_flags=3

pair_num_cutoff=3

map_len=32

rank=3

q1=/scratch/mtollis/Gopherus/workdir/final_data/Gag_1kb_R1.fq

q2=/scratch/mtollis/Gopherus/workdir/final_data/Gag_1kb_R2.fq

q=/scratch/mtollis/Gopherus/workdir/final_data/Gag_1kb_single.fq

[LIB]

avg_ins=911

reverse_seq=1

asm_flags=3

pair_num_cutoff=5

map_len=35

rank=4

q1=/scratch/mtollis/Gopherus/matepairs/paired.cat/Gag.2K.R1.fastq

q2=/scratch/mtollis/Gopherus/matepairs/paired.cat/Gag.2K.R2.fastq

[LIB]

avg_ins=2220

reverse_seq=1

asm_flags=3

pair_num_cutoff=5

map_len=35

rank=5

q1=/scratch/mtollis/Gopherus/matepairs/paired.cat/Gag.4K.R1.fastq

q2=/scratch/mtollis/Gopherus/matepairs/paired.cat/Gag.4K.R2.fastq

[LIB]

avg_ins=4446

reverse_seq=1

asm_flags=3

pair_num_cutoff=5

map_len=35

rank=6

q1=/scratch/mtollis/Gopherus/matepairs/paired.cat/Gag.10K.R1.fastq

q2=/scratch/mtollis/Gopherus/matepairs/paired.cat/Gag.10K.R2.fastq

SOAP denovo2 assembly command:

/home/mtollis/genome_assembly/SOAPdenovo2-src-r240/SOAPdenovo-127mer all -s ../Gopherus.config.file -o Gag.k47.kmer -d 2 -K 47 -N 2900000000 -p 32 1>ass.log 2>ass.err

#in this command i set the -d parameter to delete all kmers with frequency 2 or below

SOAP GapCloser command:

~/GapCloser -a Gag.k47.kmer.scafSeq -b Gag.Gapcloser.config.file -o Gag.k47.gapclosed.scaff.fa -l 104 -t 16 2>gapclose.err

B. The following is a summary of the commands used to filter the variant file (.vcf) from FreeBayes using SnpSft, and to intersect it with the *Gopherus agassizii* annotation using bedtools.

SnpSft commands:

For *Gopherus agassizii*:

cat FILE | java -jar snpSift filter "(((DP >= 20) & ( QUAL >= 30 ) & isHom( GEN[0] ) & isRef( GEN[0] )) & (isHom( GEN[1] ) & isRef( GEN[1] )) & (isHom( GEN[2] ) & isRef( GEN[2] )) & (isHom( GEN[3] ) & isVariant( GEN[3] )) & (isHom( GEN[4] ) & isVariant( GEN[4] )) & (isHom( GEN[5] ) & isVariant( GEN[5] )) & (isHom( GEN[6] ) & isVariant( GEN[6] )) & (isHom( GEN[7] ) & isVariant( GEN[7] )) & (isHom( GEN[8] ) & isVariant( GEN[8] ))) | (((DP >= 20) & ( QUAL >= 30 ) & isHom( GEN[0] ) & isVariant( GEN[0])) & (isHom( GEN[1] ) & isVariant( GEN[1])) & (isHom( GEN[2] ) & isVariant( GEN[2])) & (isHom( GEN[3] ) & isRef( GEN[3] )) & (isHom( GEN[4] ) & isRef( GEN[4] )) & (isHom( GEN[5] ) & isRef( GEN[5] )) & (isHom( GEN[6] ) & isRef( GEN[6] )) & (isHom( GEN[7] ) & isRef( GEN[7] )) & (isHom( GEN[8] ) & isRef( GEN[8] )))"

For *Gopherus morafkai*:

cat FILE | java -jar snpSift filter "(((DP >= 20) & ( QUAL >= 30 ) & isHom( GEN[3] ) & isRef( GEN[3] )) & (isHom( GEN[4] ) & isRef( GEN[4] )) & (isHom( GEN[5] ) & isRef( GEN[5] )) & (isHom( GEN[0] ) & isVariant( GEN[0] )) & (isHom( GEN[1] ) & isVariant( GEN[1] )) & (isHom( GEN[2] ) & isVariant( GEN[2] )) & (isHom( GEN[6] ) & isVariant( GEN[6] )) & (isHom( GEN[7] ) & isVariant( GEN[7] )) & (isHom( GEN[8] ) & isVariant( GEN[8] ))) | (((DP >= 20) & ( QUAL >= 30 ) & isHom( GEN[3] ) & isVariant( GEN[3])) & (isHom( GEN[4] ) & isVariant( GEN[4])) & (isHom( GEN[5] ) & isVariant( GEN[5])) & (isHom( GEN[0] ) & isRef( GEN[0] )) & (isHom( GEN[1] ) & isRef( GEN[1] )) & (isHom( GEN[2] ) & isRef( GEN[2] )) & (isHom( GEN[6] ) & isRef( GEN[6] )) & (isHom( GEN[7] ) & isRef( GEN[7] )) & (isHom( GEN[8] ) & isRef( GEN[8] )))"

For *Gopherus evgoodei*:

cat FILE | java -jar snpSift filter "(((DP >= 20) & ( QUAL >= 30 ) & isHom( GEN[6] ) & isRef( GEN[6] )) & (isHom( GEN[7] ) & isRef( GEN[7] )) & (isHom( GEN[8] ) & isRef( GEN[8] )) & (isHom( GEN[3] ) & isVariant( GEN[3] )) & (isHom( GEN[4] ) & isVariant( GEN[4] )) & (isHom( GEN[5] ) & isVariant( GEN[5] )) & (isHom( GEN[0] ) & isVariant( GEN[0] )) & (isHom( GEN[1] ) & isVariant( GEN[1] )) & (isHom( GEN[2] ) & isVariant( GEN[2] ))) | (((DP >= 20) & ( QUAL >= 30 ) & isHom( GEN[6] ) & isVariant( GEN[6])) & (isHom( GEN[7] ) & isVariant( GEN[7])) & (isHom( GEN[8] ) & isVariant( GEN[8])) & (isHom( GEN[3] ) & isRef( GEN[3] )) & (isHom( GEN[4] ) & isRef( GEN[4] )) & (isHom( GEN[5] ) & isRef( GEN[5] )) & (isHom( GEN[0] ) & isRef( GEN[0] )) & (isHom( GEN[1] ) & isRef( GEN[1] )) & (isHom( GEN[2] ) & isRef( GEN[2] )))"

Bed Tools—Intersect commands:

bedtools intersect -wb -header -a UNIQUEVARIANTFILE.vcf -b AGASSIZII_ANNOTATION.gff
