# Supplementary material for: The Agassiz’s desert tortoise genome provides a resource for the conservation of a threatened species
Source: PLoS One. 2017 May 31;12(5):e0177708. doi: 10.1371/journal.pone.0177708 (PMC5451010; doi:10.1371/journal.pone.0177708)
Supplement: S7 Dataset — (ZIP) [file pone.0177708.s021.zip › Supplementary_Dataset_7/r8s/7a_tortoise.r8s.analysis.doc]

```
#nexus
begin trees;
tree tortoise = (anoCar2:0.29304,((allMis1:0.188613,(galGal4:0.147186,(melUnd1:0.107593,(geoFor1:0.056105,taeGut2:0.048221):0.142695):0.0362343):0.20787):0.0237787,(pelSin1:0.11632,(cheMyd1:0.0383745,(chrPic1:0.0259322,gopAga1:0.0422982):0.012975):0.0330326):0.081606):0.29304);
end;

begin r8s;
#nsites
blformat lengths=persite nsites=1815350 ultrametric=no;

#define nodes
mrca sauropsida anoCar2 allMis1 taeGut2 geoFor1 melUnd1 galGal4 pelSin1 cheMyd1 chrPic1 gopAga1;
mrca testudoarch allMis1 taeGut2 geoFor1 melUnd1 galGal4 pelSin1 cheMyd1 chrPic1 gopAga1;
mrca archosauria allMis1 taeGut2 geoFor1 melUnd1 galGal4;
mrca chelonia pelSin1 cheMyd1 chrPic1 gopAga1;
mrca neognaths taeGut2 geoFor1 melUnd1 galGal4;
mrca gop-che cheMyd1 chrPic1 gopAga1;
mrca testudinoidea chrPic1 gopAga1;

#constrain nodes
fixage taxon=sauropsida age=280;
fixage taxon=archosauria age=245;
constrain taxon=chelonia max_age=250 min_age=97.4;
constrain taxon=gop-che max_age=183 min_age=36.8;
fixage taxon=testudinoidea age=74.2;
#comment out fixage taxon=testudinoidea to estimate freely

#setup analysis
divtime method=PL algorithm=TN;
set penalty=log;
showage;
describe plot=phylogram;

end;
```
